# Mutant zebrafish lacking *slc25a22a* show spontaneous seizures and respond to the anti-seizure medication valproic acid

**DOI:** 10.1242/dmm.052275

**Published:** 2025-06-20

**Authors:** So-Hyun Lee, Ting Liang, Gopalakrishnan Chandrasekaran, Jun Zhang, Seong Soon Kim, Sundareswaran Varier Parvathi, Seok Won Lee, Eun-Seo Cho, Hee-Young Shin, Young-Gyu Yoon, Jihoon Jo, Myung Ae Bae, Seok-Yong Choi, Myeong-Kyu Kim

**Affiliations:** ^1^ Department of Biomedical Sciences, Chonnam National University Medical School, Hwasun 58128, Republic of Korea; ^2^ Department of Neurology, Tangdu Hospital, Fourth Military Medical University, Xi'an 710038, China; ^3^ Therapeutics and Biotechnology Division, Korea Research Institute of Chemical Technology (KRICT), Daejeon 34114, Republic of Korea; ^4^ School of Electrical Engineering, Korea Advanced Institute of Science and Technology (KAIST), Daejeon 34141, Republic of Korea; ^5^ Department of Neurology, Chonnam National University Medical School, Gwangju 61469, Republic of Korea

**Keywords:** Epilepsy, *slc25a22a*, Anti-seizure medications, Glutamate carrier, Mitochondria

## Abstract

Epilepsy is a neurological disorder associated with abnormal neuronal activity in the central nervous system, resulting in recurrent seizures. Various anti-seizure medications (ASMs) are effective against epilepsy. However, approximately one-third of patients still do not respond to currently available ASMs either alone or in combination because the etiology of their epilepsy remains unclear. To create a novel zebrafish epilepsy model, we analyzed the exomes of 400 Korean patients with epilepsy via whole-exome sequencing. We found 39 candidate genes and investigated these genes through *in situ* hybridization and loss-of-function studies, identifying *SLC25A22*, encoding a mitochondrial glutamate carrier, as a potential epilepsy gene. Subsequently, we generated zebrafish *slc25a22a* mutants and observed that they displayed spontaneous seizures, high-voltage deflections in local field potentials, and elevated Ca^2+^ levels propagating from the forebrain to the spinal cord. Of nine ASMs tested, valproic acid (VPA) was able to suppress spontaneous seizure activities in *slc25a22a* mutant larvae, highlighting the unique anti-seizure effect of VPA in this model. Our findings provide valuable insights into the pathogenesis of epilepsy and suggest *slc25a22a* as a potential target for novel ASM development.

## Teaser

**Summary:** We generated mutant zebrafish lacking *slc25a22a*, ortholog of an epilepsy gene identified in a patient with epilepsy. The mutant exhibited key epilepsy features, including spontaneous seizures and hyperexcitability, both of which were inhibited by valproic acid.

## INTRODUCTION

Epilepsy is a chronic neurological disease affecting ∼70 million people worldwide and defined as a syndrome with at least two unprovoked seizures. Its hallmark feature is an epileptic seizure, which is a temporary occurrence of abnormally excessive or synchronous neuronal activity in the brain. Its causes are broadly classified as acquired (such as after a stroke or traumatic brain injury), infectious diseases (including meningitis, encephalitis or neurocysticercosis), autoimmune diseases and genetic variants (Singh and Trevick, 2016). To date, more than 500 genes associated with epilepsy have been identified (Devinsky et al., 2018).

Patients with epilepsy (PWEs) are primarily treated with anti-seizure medications (ASMs) to reduce or prevent seizures. Despite the availability of over 20 ASMs, however, 30-40% of patients with refractory epilepsy fail to respond to two or more ASMs. Therefore, there is an urgent need to develop novel treatment approaches and ASMs to combat epilepsy (Bialer and White, 2010).

Research on human genetic epilepsies can be improved by identifying relevant clinical traits within an experimental model system. Zebrafish (*Danio rerio*) is a widely used model organism in neurobiology as it shares many physiological and genetic properties with humans. Its central nervous system (CNS) architecture resembles that of mammals, making it a valuable tool for studying epileptogenesis and behavior during epileptic crises, and for identifying therapeutic targets and novel ASMs. Owing to its external development, zebrafish can be more easily manipulated genetically than rodents, making it useful for assessing compounds with potential effects on epileptogenesis and ASMs across various genetic models of epilepsy (D'Amora et al., 2023; Gawel et al., 2020a).

Among several zebrafish epilepsy models, the *scn1lab* mutant is the most widely used. Zebrafish *scn1lab* corresponds to the human *SCN1A* gene, which encodes the α subunit of the voltage-gated Na^+^ channel Nav1.1 (D'Amora et al., 2023; Gawel et al., 2020a; Yaksi et al., 2021). Mutant *scn1lab* zebrafish exhibit abnormal electrographic activity, hyperactivity and convulsive behaviors (Baraban et al., 2013). Through drug screening with this model, several compounds – such as fenfluramine, lorcaserin, clemizole, trazodone and synthetic cannabinoids – have been identified as potential ASMs (Whyte-Fagundes et al., 2024). The second zebrafish epilepsy model involves a mutation in the γ-aminobutyric acid (GABA) receptor α 1 subunit (*gabra1*), with fish exhibiting light-triggered seizures rather than spontaneous ones (Samarut et al., 2018). The third model has a *dep domain-containing protein 5* (*depdc5*) mutation, which leads to shorter larval body length than that of the control and reduced locomotor activity that increases upon exposure to 3 mM pentylenetetrazol (PTZ), a seizure-inducing compound (Swaminathan et al., 2018). Other zebrafish epilepsy models include those with downregulated epilepsy-related genes such as *potassium voltage-gated channel subfamily Q member 3* (*kcnq3*), *leucine-rich, glioma inactivated 1a* (*lgi1a*), *potassium inwardly rectifying channel subfamily J member 10a* (*kcnj10a*) and *syntaxin1b* (*stx1b*) (D'Amora et al., 2023). It is important to note, however, that most genetic zebrafish models are based on gene knockdowns rather than knockouts, and even knockout models exhibit a weak epilepsy phenotype; furthermore, their underlying epileptogenic mechanisms remain unclear (D'Amora et al., 2023; Griffin et al., 2021). Of note, there are several zebrafish epilepsy models that show hypoactivity (Gawel et al., 2024, 2020b; Griffin et al., 2021).

We identified mutation of *SLC25A22* [also called glutamate carrier 1 (*GC1*)], through exome sequencing of 400 Korean PWEs, developed a zebrafish *slc25a22a* mutant model using CRISPR-Cas9 and found that valproic acid (VPA) suppresses seizures in this model, suggesting Slc25a22a as a potential target for development of epilepsy treatments.

## RESULTS

### Identification of candidate epilepsy-related genes

Through whole-exome sequencing (WES) of 400 Korean PWEs, we identified 82 variants of 39 candidate genes, with minor allele frequency (MAF) being zero or not being registered in gnomAD or the Korean Reference Genome Database (KRGDB) as of 2017 (Table S1). Of these candidate genes, we excluded from further study genes that had been investigated extensively elsewhere. To test whether these genes are expressed in the CNS, we performed whole-mount *in situ* hybridization (WISH) and found that most of them are indeed expressed in the brain (Fig. S1). To investigate whether their variants are associated with epilepsy, we downregulated these genes in zebrafish using antisense oligonucleotide morpholinos (MOs) and observed hyperactivity in *slc25a22a* and *slc25a22b* morphants, but not in other morphants, when exposed to 1 mM PTZ (Figs S2 and S3). Homozygous variants of human *SLC25A22* were reported to cause developmental and epileptic encephalopathy 3 [Online Mendelian Inheritance in Man (OMIM) #609304] (Molinari et al., 2009, 2005; Poduri et al., 2013).

**Fig. 1.**
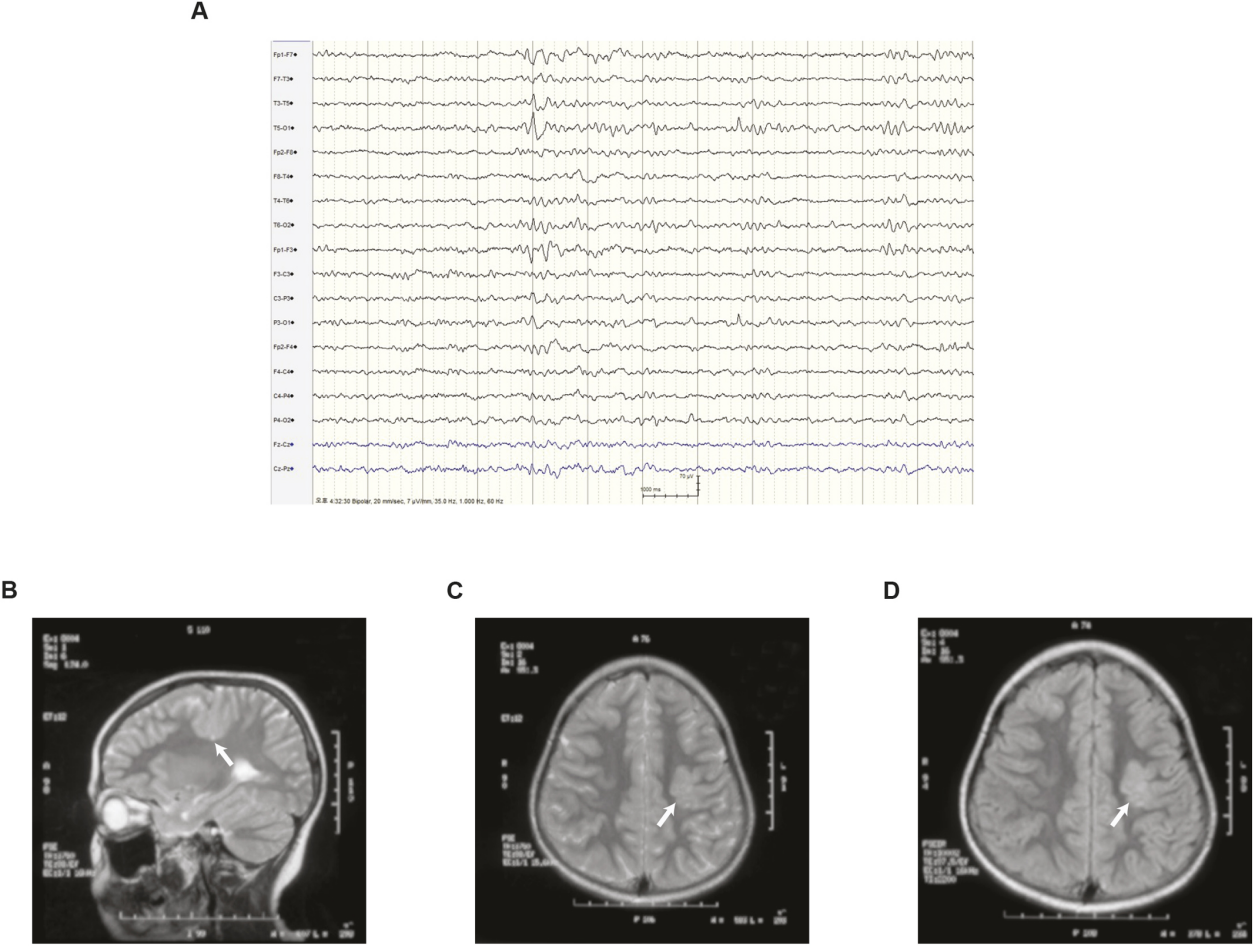
**Clinical profiles of an epilepsy patient with *SLC25A22* mutation.** (A) An electroencephalogram of the patient at the age of 24. (B-D) Brain magnetic resonance imaging of the patient at the age of 9. T2-weighted sagittal (B), axial (C) and fluid-attenuated inversion recovery (FLAIR; D) images are shown. White arrows indicate the left cortical heterotopia.

Of the aforementioned 400 Korean PWEs, a heterozygous variant of *SLC25A22* – NM_001191060.1: c.194T>C; NP_001177989.1: p.(Met65Thr) – was found in a 26-year-old woman. WES of her mRNA did not show any other pathogenic variants of known epilepsy genes. Her seizures first occurred at the age of 3 years and evolved from focal aware sensory seizures (electrical sensations around the nose) to bilateral tonic–clonic seizures through focal aware motor seizures (clonic movements of the right hand). She was treated first with carbamazepine. However, focal sensory or motor seizures persisted until age 14. She was transferred to our epilepsy clinic at the age of 16 and showed only sensory seizures. Her electroencephalogram at the age of 24 displayed an isolated epileptiform discharge in the left temporal area (Fig. 1A). Brain magnetic resonance imaging performed at the age of 9 showed cortical heterotopia in the left frontal lobe (Fig. 1B-D). She did not show clinical features of epileptic encephalopathy and responded well to ASMs: a combination of zonisamide and lamotrigine.

**Fig. 2.**
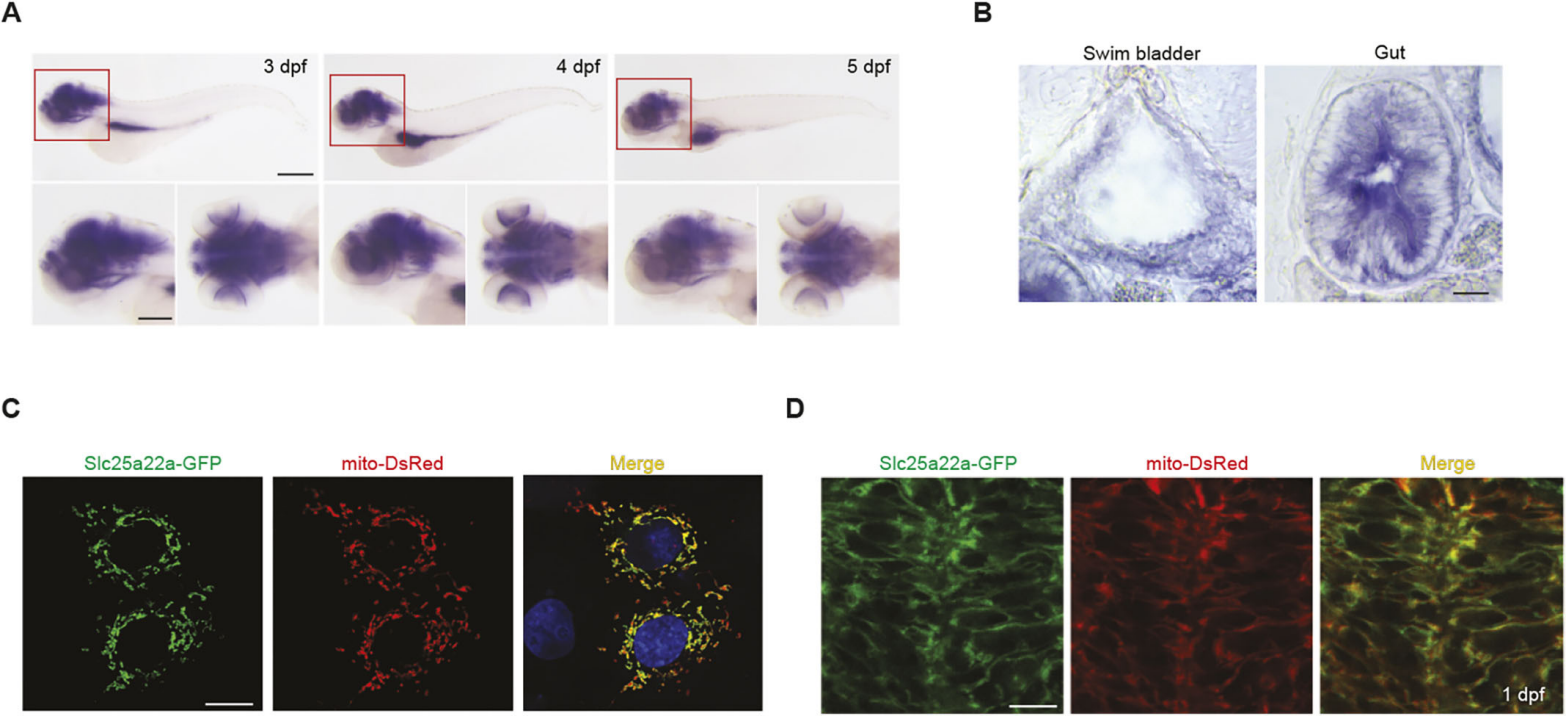
**Zebrafish *slc25a22a* is expressed in the brain, intestine and swim bladder, and Slc25a22a is localized in mitochondria of the forebrain.** (A) Upper row: wild-type (WT) zebrafish larvae at 3-5 days post-fertilization (dpf) were probed with the *slc25a22a* riboprobe. Lateral view anterior to the left. The boxed areas are magnified in the respective panels in the lower row. Left, lateral view; right, dorsal view anterior to the left. Scale bars: 300 μm (upper); 100 μm (lower). (B) Cross-section images of the intestine and swim bladder of larvae at 5 dpf in A. Scale bar: 10 μm. (C) Co-transfection of COS-7 cells with the plasmid encoding GFP-tagged zebrafish Slc25a22a and the plasmid encoding mitochondrially targeted DsRed. Scale bar: 20 μm. (D) GFP-tagged *slc25a22a* RNAs were microinjected into a one-cell-stage *Tg(mito:dsred)* embryo, and its forebrain region was imaged at 1 dpf. Scale bar: 10 μm.

Zebrafish has two human SLC25A22 orthologs: Slc25a22a and its paralog Slc25a22b. Slc25a22a has higher similarity in amino acid sequences to its human and mouse orthologs than does Slc25a22b (79% versus 73%). *slc25a22a* is expressed in the brain, intestine and swim bladder, whereas *slc25a22b* is expressed more broadly throughout the organism (Fig. 2A,B; Fig. S1). *slc25a22a* is expressed throughout the brain, not restricted to the forebrain. We chose *slc25a22a* over *slc25a22b* for further investigation because the expression of the former is more restricted to the brain than that of the latter. To determine its subcellular localization, we co-transfected COS-7 cells with the plasmid encoding GFP-tagged zebrafish Slc25a22a and the plasmid encoding mitochondrially targeted DsRed, noting mitochondrial-specific localization of Slc25a22a (Fig. 2C). This finding was corroborated in *Tg(mito:dsred)* zebrafish embryos, in which DsRed was expressed selectively in mitochondria: microinjection of GFP-tagged *slc25a22a* RNA into the embryos resulted in the colocalization of GFP with DsRed in the forebrain (Fig. 2D). Hence, these findings indicate that Slc25a22a is localized in mitochondria in the zebrafish forebrain.

### Generation of *slc25a22a* mutant by CRISPR-Cas9 technology

**Fig. 3.**
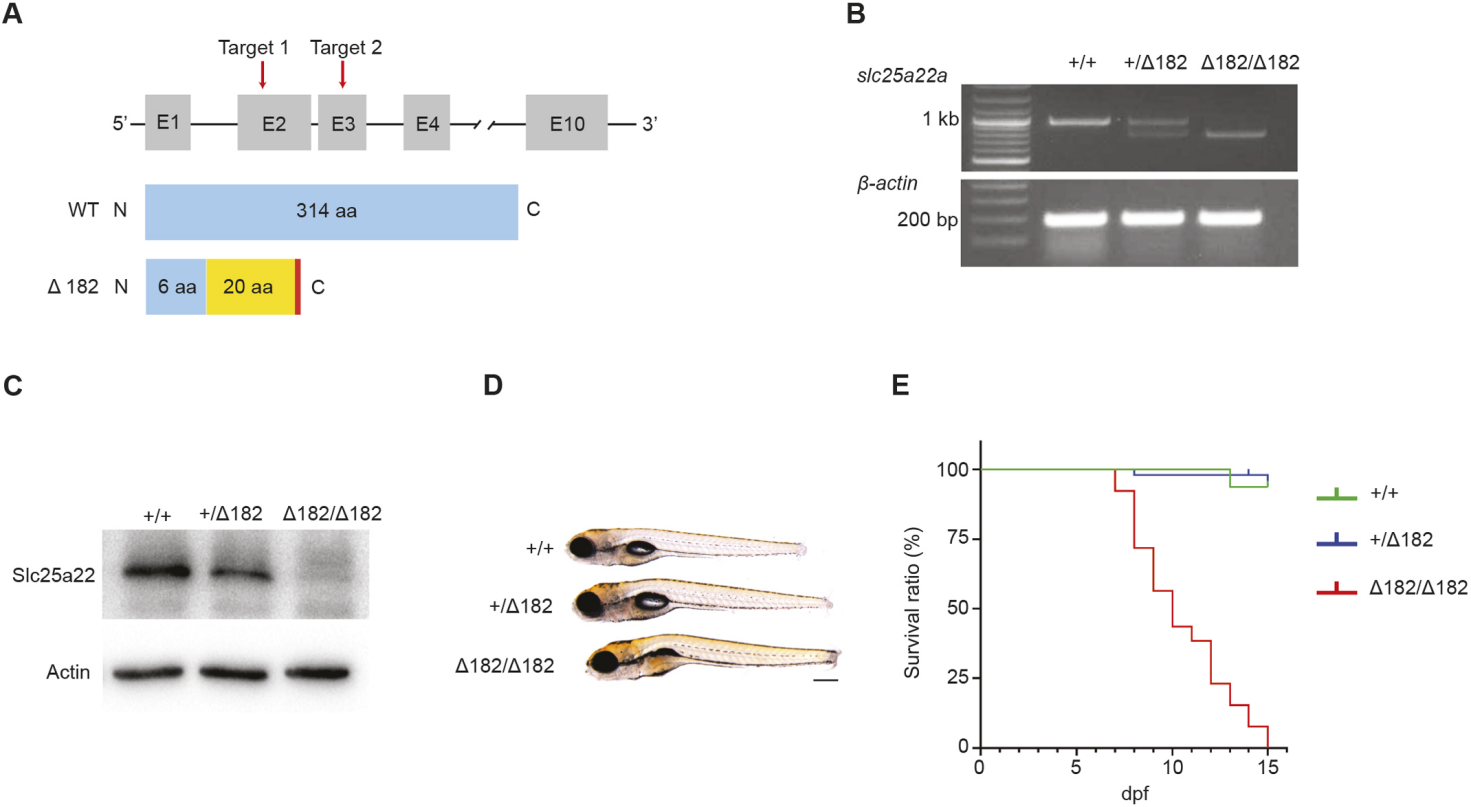
***slc25a22a^Δ5/Δ5^* and *slc25a22a^Δ182/Δ182^* mutants are generated with CRISPR-Cas9 technology.** (A) Upper: schematic of the zebrafish *slc25a22a* locus and location of CRISPR-Cas9 targets (red arrows); lower: schematic of Slc25a22a proteins translated in WT and *slc25a22a^Δ182/Δ182^* mutants generated with the CRISPR-Cas9 technology. The blue and yellow boxes indicate WT and introduced amino acids (aa), respectively, and the red bar represents a premature stop codon. (B) RT-PCR analysis of the targeted *slc25a22a* region in the indicated alleles. mRNAs were harvested from zebrafish larvae at 5 dpf and amplified with gene-specific primers. β-Actin primers were used for loading control. (C) Western blot analysis of Slc25a22 proteins in 5 dpf larvae with the indicated alleles. Actin protein was used as a loading control. (D) Larvae with the indicated alleles at 5 dpf were imaged under a light microscope. Lateral view anterior to the left. Scale bar: 200 μm. (E) *slc25a22a^+/+^* (*n*=16), *slc25a22a^+/−^* (*n*=49) and *slc25a22a^−/−^* (*n*=39) embryos were raised to 15 dpf, and their respective Kaplan–Meier survival rate curves were generated.

To investigate a role for Slc25a22a in epilepsy, we used CRISPR-Cas9 technology targeting exons 2 and 3 of *slc25a22a*, resulting in deletion of 5 bp or 182 bp. These mutants were designated as *slc25a22a^Δ5^* and *slc25a22a^Δ182^*, respectively (Fig. 3A; Fig. S4A). These frameshift mutations introduced a premature stop codon that led to nonsense-mediated decay and deleted WT Slc25a22a proteins (Fig. 3B,C; Fig. S4B,C, Fig. S5). The morphology of both homozygous mutants appeared normal at 5 days post-fertilization (dpf) except for an uninflated swim bladder, whereas both heterozygous mutants were visually indistinguishable from *slc25a22a^+/+^* [wild-type (WT)] siblings (Fig. 3D; Fig. S4D). As *slc25a22a* was expressed in the swim bladder (Fig. 2B), which was not inflated in the homozygous mutants, we reasoned that Slc25a22a may be implicated in development and/or inflation of zebrafish swim bladder. Both homozygous mutants' larvae had died by 15 dpf, with mortality appearing at ∼8-11 dpf (Fig. 3E; Fig. S4E).

### *slc25a22a^Δ5/Δ5^* and *slc25a22a^Δ182/Δ182^* mutants display spontaneous seizures

**Fig. 4.**
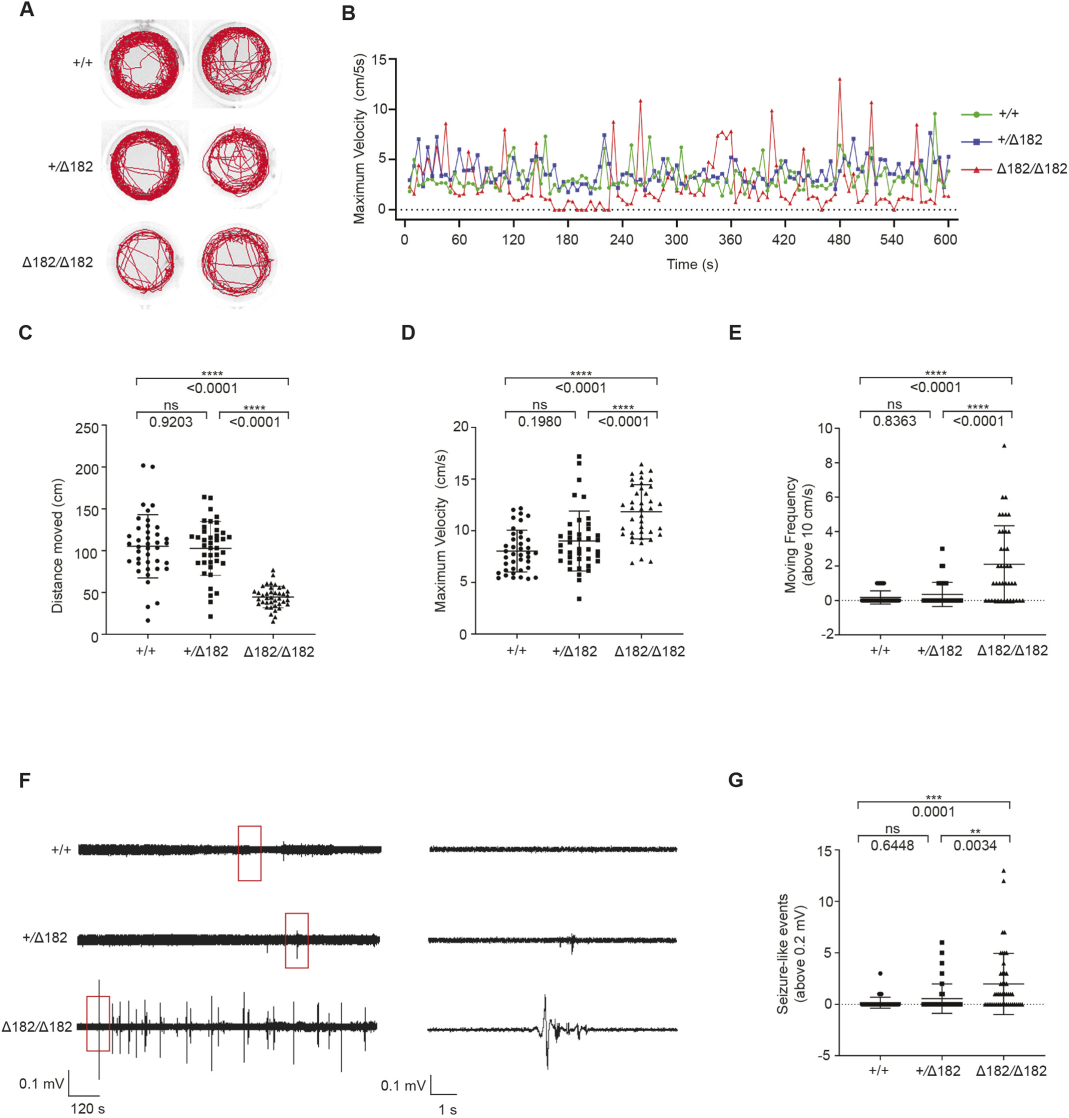
***slc25a22a^Δ182/Δ182^* mutants display spontaneous seizures.** (A) Representative locomotor activity traces of *slc25a22a^+/+^*, *slc25a22a^+/Δ182^* and *slc25a22a^Δ182/Δ182^* larvae at 5 dpf for 10 min. (B) Representative traces of swim maximum velocity (cm/5 s) in *slc25a22a^+/+^* (green), *slc25a22a^+/Δ182^* (blue) and *slc25a22a^Δ182/Δ182^* (red) larvae at 5 dpf for 10 min. (C-E) The total swimming distance (C), maximum swimming velocity (D) and moving frequency (over 10 cm/s) (E) assessed for 10 min in *slc25a22a^+/+^*, *slc25a22a^+/Δ182^* and *slc25a22a^Δ182/Δ182^* larvae at 5 dpf*.* Data are presented as mean±s.d. ns, not significant; ****P<0.0001 by one-way ANOVA with Tukey's honest significant difference (HSD) post hoc test (*n*=40 embryos per each group). (F) Representative local field potential (LFP) recordings of *slc25a22a^+/+^* (*n=*40), *slc25a22a^+/Δ182^* (*n=*40) and *slc25a22a^Δ182/Δ182^* larvae (*n*=47) at 5 dpf. Boxed areas are magnified in the respective right panel. (G) Frequency of spikes above 0.2 mV was measured in *slc25a22a^+/+^*, *slc25a22a^+/Δ182^* and *slc25a22a^Δ182/Δ182^* larvae at 5 dpf*.* Data are presented as mean±s.d. ns, not significant; **P< 0.01 and ***P< 0.001 by one-way ANOVA with Tukey's HSD post hoc test. Of note, the experiments shown in F and G were performed simultaneously with those in Fig. 6C,D using shared +/+ and Δ182/Δ182 groups.

To check whether *slc25a22a^Δ5/Δ5^* and *slc25a22a^Δ182/Δ182^* show a spontaneous seizure-like phenotype, we examined the locomotor behavior of 5 dpf larvae using automated behavioral tracking. As reported previously (Hotz et al., 2022), WT zebrafish moved forward in a single motion and then slowed down or stopped moving. However, mutant larvae occasionally displayed convulsive twitching and swim bursts while swimming, followed by swirling around their body axes (Fig. 4A,B; Fig. S6A,B, Movie 1). Compared to their siblings, both mutants exhibited abnormal locomotor behaviors, such as decreased distance moved (Fig. 4C; Fig. S6C), significantly higher maximum swimming velocities (Fig. 4D; Fig. S6D) and occasional swim bursts (moving frequency above 10 cm/s) (Fig. 4E; Fig. S6E). These findings were also observed in *slc25a22a* morphants (Fig. S3), ruling out any CRISPR-Cas9 off-target effects. These behaviors are reminiscent of those shown in the established epilepsy models (Hotz et al., 2022). Because both *slc25a22a^Δ5/Δ5^* and *slc25a22a^Δ182/Δ182^* mutants exhibited comparable spontaneous seizures, we conducted subsequent experiments using *slc25a22a^Δ182/Δ182^* larvae only. The *slc25a22a^Δ182/Δ182^* mutants are hereafter referred to as *slc25a22a* mutants. To verify the occurrence of seizure activities in these mutants, we recorded local field potentials (LFPs) in the anterior forebrain of 5 dpf larvae. The mutant larvae displayed higher-voltage deflections in LFPs (above 0.2 mV) than their WT and heterozygous siblings (Fig. 4F,G). Collectively, these results suggest that *slc25a22a^−/−^* larvae show spontaneous seizure activities.

### *slc25a22a* mutant larvae exhibit an increase in Ca^2+^ levels propagating from the forebrain to the spinal cord

**Fig. 5.**
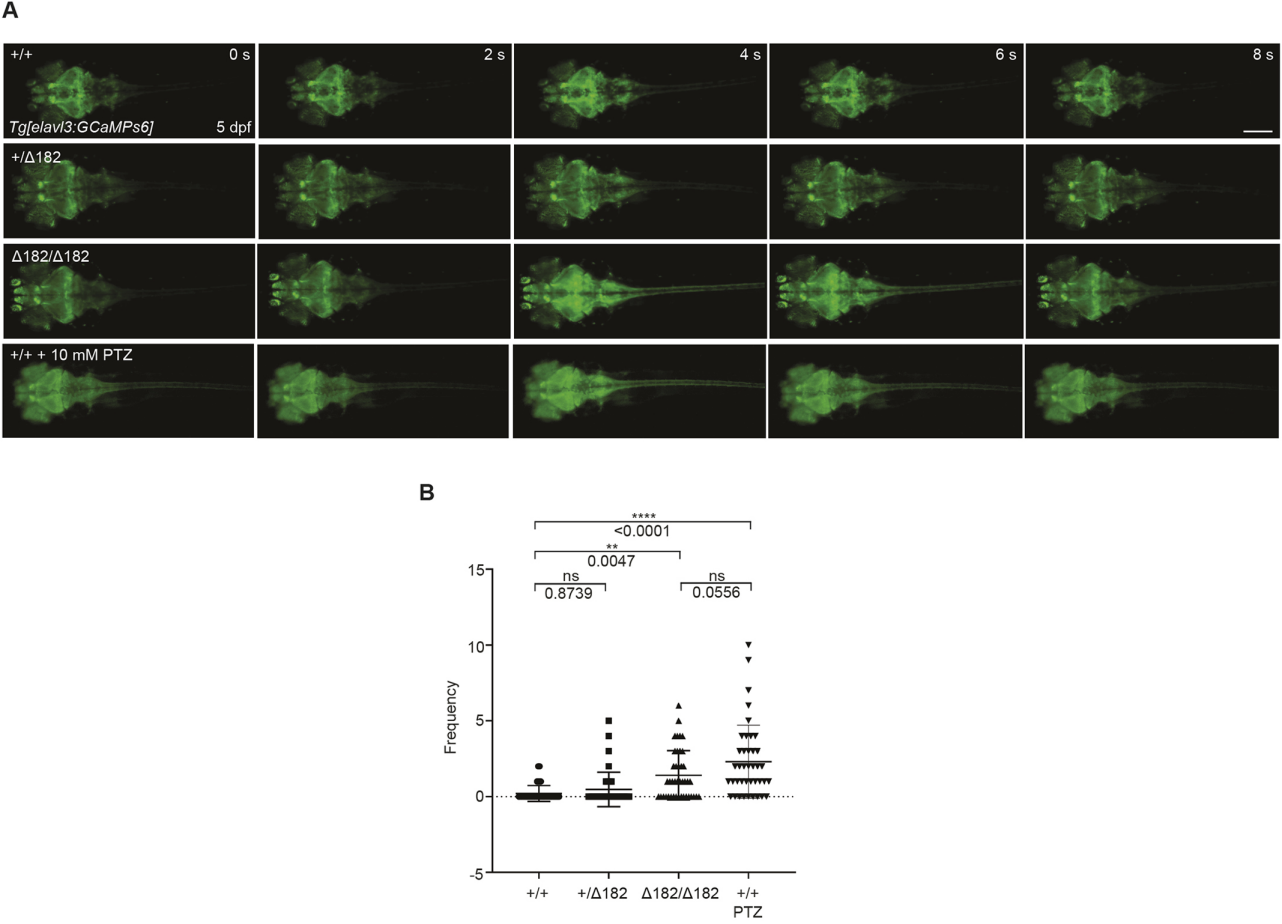
***slc25a22a^Δ182/Δ182^* larvae exhibit elevated Ca^2+^ propagation from the midbrain to the spinal cord.** (A) *Tg(elavl3:Gal4,UAS:GCaMP6s)* embryos at 5 dpf expressing GCaMP6s, a Ca^2+^ indicator, in mature neurons were subjected to time-lapse imaging for 20 min. WT larvae were exposed to 10 mM pentylenetetrazol (PTZ) for 30 min before the imaging. Scale bar: 300 μm. (B) Frequency of Ca^2+^ propagation from the midbrain to the spinal cord was measured for 20 min. Data are presented as mean±s.d. ns, not significant; **P<0.01 and ****P<0.0001 by one-way ANOVA with Tukey's HSD post hoc test (*slc25a22a^+/+^*, *n*=39; *slc25a22a^+/Δ182^*, *n*=40; *slc25a22a^Δ182/Δ182^*, *n*=42; WT+PTZ, *n*=40). Of note, the experiments shown in A and B were performed simultaneously with those in Fig. 6E,F using shared +/+ and Δ182/Δ182 groups.

Elevated Ca^2+^ levels within neurons are crucial in initiating and propagating seizure activity. Whole-brain neuronal synchrony increases during PTZ-induced seizures in zebrafish (Burrows et al., 2020). To investigate whether *slc25a22a* mutant larvae display altered neuronal Ca^2+^ levels, we recorded neuronal Ca^2+^ levels using *Tg(elavl3:Gal4,UAS:GCaMP6s)*, which expresses the Ca^2+^ indicator GCaMP6s in mature neurons (Giacomotto et al., 2016; Robles et al., 2014). The mutant larvae exhibited elevated Ca^2+^ levels propagating from the forebrain to the spinal cord, which was also noted in WT larvae exposed to 10 mM PTZ. However, this was rarely observed in WT and heterozygous siblings without PTZ exposure (Fig. 5A,B; Movies 2-5). Thus, the propagation of increased Ca^2+^ levels may induce sudden neuronal activity in *slc25a22a* mutant larvae.

### VPA can suppress spontaneous seizure activities in *slc25a22a* mutant larvae

We next investigated whether known ASMs could inhibit a spontaneous seizure phenotype in *slc25a22a* mutant larvae. To find the highest non-toxic concentrations (maximum tolerated concentrations) of each drug, we treated the WT larvae with several commonly used ASMs at concentrations ranging from 25 μM to 500 μM and observed their behavior. The treated larvae exhibited no significant difference in movement distance over a 10-min period compared to untreated larvae at the following concentrations: VPA, levetiracetam (LEV) and topiramate (TPM), 300 μM; oxcarbazepine (OXC), 200 μM; lamotrigine (LTG), 50 μM; lacosamide (LCM), 30 μM; carbamazepine (CBZ), 25 μM ; tiagabine (TGB), 10 μM; and vigabatrin (VGB), 10 mM and 20 mM (Fig. S7). We then assessed the locomotor behavior of 5 dpf mutants exposed to each drug at the aforementioned concentrations for 2 h.

**Fig. 6.**
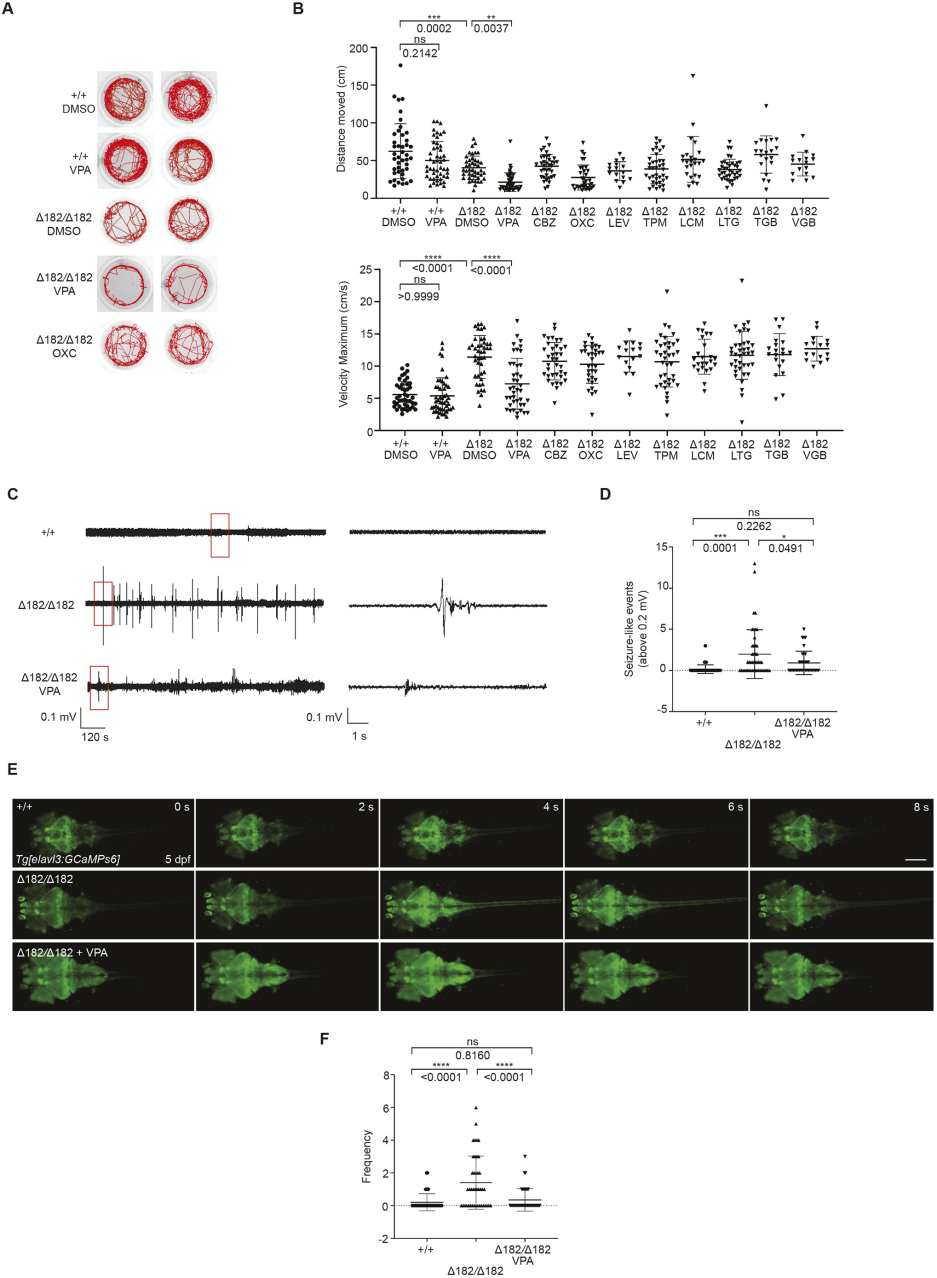
**Valproic acid can suppress the spontaneous seizure activities in *slc25a22a^Δ182/Δ182^* larvae.** (A) Representative locomotor activity traces recorded for 10 min in 5 dpf *slc25a22a^+/+^* and *slc25a22a^Δ182/Δ182^* larvae exposed to 300 μM valproic acid (VPA) and 50 μM oxcarbazepine (OXC). DMSO, dimethyl sulfoxide. (B) *slc25a22a^Δ182/Δ182^* larvae at 5 dpf were exposed to the indicated anti-seizure medications (ASMs), and their total swimming distance and velocity maximum were measured. Data are presented as mean±s.d. ns, not significant; **P<0.01 and ***P<0.001 and ****P<0.0001 by one-way ANOVA with Tukey's HSD post hoc test. No statistically significant differences were noted between Δ182+DMSO and Δ182+all ASMs except for VPA. *slc25a22a^+/+^* or *slc25a22a^Δ182/Δ182^* + DMSO, *n*=43 each; *slc25a22a^Δ182/Δ182^* + VPA, *n*=41; *slc25a22a^Δ182/Δ182^* + carbamazepine (CBZ) or lamotrigine (LTG), *n*=38 each; *slc25a22a^Δ182/Δ182^* + OXC, *n*=16; *slc25a22a^Δ182/Δ182^* + lacosamide (LCM), *n*=24; *slc25a22a^Δ182/Δ182^* + levetiracetam (LEV), *n*=37; *slc25a22a^Δ182/Δ182^* + topiramate (TPM), *n*=33; *slc25a22a^Δ182/Δ182^* + tiagabine (TGB), *n*=20; *slc25a22a^Δ182/Δ182^* + vigabatrin (VGB), *n*=16. (C) Representative recordings of LFP in 5 dpf *slc25a22a^+/+^*, *slc25a22a^Δ182/Δ182^* and *slc25a22a^Δ182/Δ182^* larvae treated with VPA. Boxed areas are magnified in the respective right panel. (D) Frequency of spikes above 0.2 mV was measured in the larvae in C*.* Data are presented as mean±s.d. ns, not significant; *P<0.05 and ***P<0.001 by one-way ANOVA with Tukey's HSD post hoc test (*slc25a22a^+/+^*, *n*=40; *slc25a22a^Δ182/Δ182^*: *n*=47, *slc25a22a^Δ182/Δ182^*+VPA, *n*=36). (E) Representative images of *Tg(elavl3:Gal4,UAS:GCaMP6s)* larvae at 5 dpf with the indicated genotypes subjected to time-lapse imaging for 20 min. Scale bar: 300 μm. (F) Frequency of Ca^2+^ propagation from the midbrain to the spinal cord was measured in the larvae in E for 20 min. Data are presented as mean±s.d. ns, not significant; ****P<0.0001 by one-way ANOVA with Tukey's HSD post hoc test (*slc25a22a^+/+^*, *n*=39; *slc25a22a^Δ182/Δ182^*, *n*=42; *slc25a22a^Δ182/Δ182^* + VPA, *n*=43). Of note, the experiments shown in C and D, and the experiments shown in E and F, were performed simultaneously with those in Fig. 4F,G and Fig. 5A,B, respectively, using shared +/+ and Δ182/Δ182 groups.

VPA treatment restored the maximum swimming velocity, although not to WT levels. However, the other ASMs did not restore the overall distance moved (Fig. 6A,B). To confirm the VPA-induced decrease in seizure activities, we recorded LFPs in the anterior forebrain of 5 dpf larvae upon VPA treatment and found that VPA indeed reduced the high-voltage deflections in LFPs in the mutants (Fig. 6C,D). Furthermore, VPA decreased Ca^2+^ propagation from the forebrain to the spinal cord in the mutants with the *Tg(elavl3:Gal4,UAS:GCaMP6s)* background as well (Fig. 6E,F; Movie 6). Collectively, these findings indicate that, of the ASMs tested, only VPA can suppress spontaneous seizure activities in *slc25a22a* mutant larvae.

## DISCUSSION

In this study, we observed traits of *slc25a22a* mutant zebrafish that mirror key features of human epilepsy, including spontaneous seizures and electrophysiological hyperexcitability. In addition, these two epilepsy traits were inhibited by VPA treatment. These findings highlight the relevance of the *slc25a22a* mutant zebrafish model for investigating the mechanisms underlying the seizures associated with the absence of Slc25a22.

Glutamate in the chemical synaptic cleft that is released from glutamatergic neurons can bind to ionotropic and metabotropic glutamate receptors on postsynaptic neurons. This glutamate is removed from the synaptic cleft through reuptake by excitatory amino acid transporters (EAATs) on neurons and astrocytes to prevent neuronal overexcitation. In astrocytes, glutamate is metabolized by glutamine synthetase (GS) into glutamine**,** which is then transferred back to glutamatergic neurons. This process completes the glutamine–glutamate cycle, which is critical for maintaining the balance between excitation and inhibition in the nervous system (Barker-Haliski and White, 2015; Benjamin and Quastel, 1975; Rowley et al., 2012; Sarlo and Holton, 2021; van den Berg and Garfinkel, 1971; Zhou and Danbolt, 2014). Therefore, alterations in this cycle could elicit epileptogenesis. For example, an increase in intracellular glutamate concentration reduces the reuptake of extracellular glutamate (Barbour et al., 1991; Otis and Jahr, 1998; Tzingounis and Wadiche, 2007). Dysfunction of a couple of proteins has been implicated in high intracellular glutamate concentration. First, the lack of GS causes the accumulation of glutamate in astrocytes, which is associated with epilepsy (Eid et al., 2004; Haberle et al., 2005, 2011; Laake et al., 1995; Perez et al., 2012). Second, the downregulation of Slc25a22, a carrier that transports glutamate into mitochondria, in rat primary astrocytes results in the accumulation of intracellular glutamate, potentially initiating hyper-excitability in the neuronal network (Goubert et al., 2017).

Colleaux, Molinari, Salih, Walsh and colleagues identified *SLC25A22* as being linked to epilepsy of infancy (Molinari et al., 2009, 2005; Poduri et al., 2013). Because SLC25A22 (also known as GC1) plays an important role in mitochondrial energy production and neurotransmitter regulation, mutation of SLC25A22 can cause mitochondrial dysfunction (Andre et al., 2021). Mitochondrial dysfunction can trigger epilepsy through various mechanisms. First, it can lead to deficiency in energy production, which is critical in high-energy-demanding tissues such as the brain, potentially triggering seizures. Second, it can induce oxidative stress, causing inflammation and nerve cell damage, which are linked to seizures. Third, it can disrupt Ca^2+^ regulation, which is crucial for neuronal function and can trigger seizure. Lastly, mitochondrial dysfunction can elicit neurotransmitter imbalance, furthering seizure susceptibility (Zsurka and Kunz, 2015). Previous studies have shown that SLC25A22 inactivation in astrocyte mitochondria decreased nicotinamide adenine dinucleotide (NAD^+^) and ATP levels, and resulted in accumulated intracellular glutamate, which may be responsible for the increased intracellular Ca^2+^, altered neuronal synchronicity and epilepsy in SLC25A22-deficient patients (Goubert et al., 2017; Green et al., 2021; Mark et al., 2001). Furthermore, despite reports linking dysfunction of the glutamate carrier to epilepsy, no genetic mouse or zebrafish models mimicking this condition have been reported. This may account for, at least in part, poorly understood mechanisms underlying epilepsy associated with *SLC25A22* variants (Barker-Haliski and White, 2015). We believe that our zebrafish model of mitochondrial glutamate carrier-associated epilepsy could help uncover molecular mechanisms of this type of epilepsy.

Although numerous zebrafish epilepsy models have been developed, most of them showed (1) light- or chemical-triggered seizures rather than spontaneous seizures, (2) weak seizure phenotypes or (3) concomitant severe developmental defects (Dogra et al., 2023; Hotz et al., 2022; Samarut et al., 2018; Swaminathan et al., 2018; Tanwar et al., 2019). Among these models, the *scn1lab* zebrafish mutant is one of the most widely used to screen for ASM activities (D'Amora et al., 2023; Sourbron et al., 2019; Tiraboschi et al., 2020; Weuring et al., 2020). Nevertheless, we believe that our *slc25a22a* mutant could be an alternative epilepsy model based on the following reasons. First, 1 mM VPA, but not 250 μM VPA, decreased the seizure activities of *scn1lab* zebrafish mutants (Li et al., 2021), whereas 300 μM VPA decreased the seizure activities of *slc25a22a* mutants (Fig. 3C, Fig. 4B and Fig. 6A). The ability to suppress seizures with lower drug concentrations can promote drug safety, as high drug concentrations can pose a higher risk of side effects (Goldenberg, 2010). Second, *slc25a22a* mutants, but not *scn1lab* mutants, show almost no movement after a swim burst, which is reminiscent of human seizure (Friedman and Sharieff, 2006). Third, the *slc25a22a* mutant operates through a mechanism that differs from that of the *scn1lab* mutant. The *slc25a22a* mutant is expected to affect mitochondrial function, whereas the *scn1lab* mutant alters voltage-gated Na^+^ channel activity. As such, the *slc25a22a* mutant could perfectly complement the *scn1lab* mutant. Finally, the current study on *slc25a22a* mutant zebrafish investigated the relationship between seizures and Ca^2+^ levels, which has not been reported to date. Ca^2+^ is essential for regulation of various neuronal functions, such as neurotransmitter release, neuronal signal transmission and overall cell activity. Therefore, Ca^2+^ levels should be properly regulated to maintain brain functions. During seizures, the excessive activation of Ca^2+^ channels rapidly increases Ca^2+^ levels, which can promote the hyperactivation of neurons and the occurrence of seizures (Delorenzo et al., 2005). The *slc25a22a* mutants in the current study exhibited an increase in Ca^2+^ levels propagating from the forebrain to the spinal cord, which was blunted by VPA treatment. Of note, the current study is the first to show that an ASM can decrease Ca^2+^ propagation in a zebrafish model of epilepsy. Taken together, our findings suggest that the *slc25a22a* zebrafish model could complement the *scn1lab* model. A zebrafish model of epilepsy that can reproduce various epilepsy traits would be of great use to study various aspects of epilepsy pathophysiology and to test potential therapeutic interventions (D'Amora et al., 2023; Gawel et al., 2020a; Yaksi et al., 2021). As such, the *slc25a22a* mutant could help with delineation of the molecular pathomechanism(s) of mitochondria-associated epilepsy and development of efficient drugs. However, as our *slc25a22a* mutants do not survive into adulthood, this mutant could not be used to study adult epileptogenesis. This is the limitation of our zebrafish model.

Our research revealed the potential of VPA for treating mitochondria-associated seizures. In our study, we tested nine commonly used ASMs: VPA, CBZ, OXC, LEV, TPM, LCM, LTG, TGB and VGB (Sills and Rogawski, 2020). CBZ and OXC act as Na^+^ channel blockers (Dogra et al., 2023; Lin et al., 2021), whereas LEV regulates neurotransmitter release (Abou-Khalil, 2008). TPM modulates various channels and receptors, thereby showing neuroprotective properties (Angehagen et al., 2004), and LCM and LTG act on Na^+^ channels and ion channels, respectively (Costa and Vale, 2023; Kellinghaus, 2009). VGB acts as an irreversible suicide inhibitor of GABA transaminase, and TGB blocks GABA reuptake into neurons and glia (Treiman, 2001). Among these drugs, VPA stood out for its ability to reduce spontaneous seizures in the *slc25a22a* mutants (Fig. 6A,B). This mutant is most likely deficient in mitochondrial glutamate transport (Molinari et al., 2009, 2005; Poduri et al., 2013), resulting in accumulation of the excitatory neurotransmitter glutamate in astrocytes, which might initiate hyper-excitability in the neuronal network (Goubert et al., 2017). One of the anti-seizure mechanisms of VPA involves increasing the levels of GABA, an inhibitory neurotransmitter (Chateauvieux et al., 2010; Ghodke-Puranik et al., 2013). Hence, the anti-seizure effect of VPA on the *slc25a22a* mutants may be attributed to its ability to enhance GABA levels. As such, this mode of action could support the use of VPA in patients with a pathogenic variant in *SLC25A22*. However, zebrafish exposed to 25 μM VPA for 10 h or 500 μM VPA for 3 days were reported to exhibit neurotoxic effects, as well as social interaction deficits, anxiety, hyperactivity and sleep-like behavior (Chen et al., 2018; DeOliveira-Mello et al., 2023). Therefore, it is conceivable that the anti-seizure-like effect of VPA in our model is caused by the aforementioned side effects. We believe, however, that this is not likely because WT larvae treated with 300 μM VPA for 2 h did not exhibit such side effects. Of note, VPA treatment significantly decreased discharges in the electroencephalogram of zebrafish larvae overexpressing *scn1lab* (Silvennoinen et al., 2022).

Why did VPA treatment not recover the movement distance of *slc25a22a* mutants? In zebrafish models of epilepsy, the movement distances vary as follows: (1) in PTZ-triggered seizures, the movement distance was enhanced (D'Amora et al., 2023); (2) *scn1lab* mutants showed either increased or similar total distance traveled compared to that of WT siblings (Baraban et al., 2013; Griffin et al., 2021); (3) a recent report on *eaat2a* mutants showed reduced swimming distance (Hotz et al., 2022), comparable to that in our *slc25a22a* mutants; and (4) the efficacy of ASMs was assessed in terms of maximum velocity (Baraban et al., 2013; Whyte-Fagundes et al., 2024). This variation suggests that the effects of epilepsy traits on movement distance in zebrafish models of epilepsy warrant further research.

In contrast to previously reported cases (Lemattre et al., 2019; Molinari et al., 2009, 2005; Poduri et al., 2013), our patient had a heterozygous *SLC25A22* variant and experienced her first seizure at the age of 3. Although we did not identify any pathogenic variants in known epilepsy genes through WES, we could not rule out the possibility that our patient's epilepsy might be caused by a variant(s) in a novel epilepsy gene.

A number of variants of *SLC25A22* have been found in PWEs: p.(Pro206Leu) (Molinari et al., 2005), p.(Gly236Trp) (Molinari et al., 2009), p.(Gly110Arg) (Poduri et al., 2013), p.(Thr56Pro), p.(Glu79Lys), p.(Val249Glu), p.(Ala296Thr) (Reid et al., 2017), p.(Cys246Arg), p.(Glu79Gln) (Giacomini et al., 2019), p.(Ala272Glnfs*144), p.(Arg273Lys) (Lemattre et al., 2019), p.(Glu279Glyfs*138) (Andre et al., 2021) and p.(Lys33Glu) (Nicotera et al., 2021). In addition, these variants are associated with a diverse spectrum of phenotypes. However, their genotype–phenotype correlation remains unclear. In patients with *SLC25A22* variants, seizures typically occurred early in life, and, in severe cases, developmental delays were observed (Andre et al., 2021). Additionally, patient fibroblasts have shown extensive vacuolation containing neutral lipids and phospholipids, indicating the presence of metabolic abnormalities (Reid et al., 2017). In our study, we observed the expression of the *slc25a22a* gene not only in the brain but also in the intestine of zebrafish (Fig. 2A,B). This finding suggests that metabolic abnormalities, similar to those noted for patients with *SLC25A22* variants, also occur in zebrafish. The determination of the underlying mechanisms of these metabolic abnormalities could provide insights into the pathogenesis of epilepsy and the molecular mechanisms underlying broader issues related to metabolic dysfunction. By investigating the shared pathways involved in both epilepsy and metabolic abnormalities, we could identify new therapeutic targets that could have implications for treating a range of disorders beyond epilepsy alone. This broader perspective underscores the potential significance of our findings for both basic research and clinical applications in the field of neurological and metabolic disorders. Thus, our *slc25a22a* mutant zebrafish model could aid in the discovery of the molecular mechanism(s) by which *slc25a22a* mutation triggers epilepsy and the development of new ASMs.

## MATERIALS AND METHODS

### Ethics approval statement

Zebrafish experiments described in this study were approved by the Animal Experiment Ethics Committee of the Center for Convergence of Life Science, Chonnam National University (CNU IACUC-H-2023-25). The human study protocol was approved by the Institutional Review Board at Chonnam National University Hospital (CNUH-2016-028) and was conducted in accordance with the Declaration of Helsinki 1975. All patients or their guardians gave written informed consent to participate in this study.

### WES of 400 Korean PWEs

Included in the 400 Korean PWEs were consecutive patients with an established clinical diagnosis of epilepsy (Fisher et al., 2014) who had been under the care of epilepsy specialists for at least 2 years. Excluded were those with a definite family history of epilepsy in first- or second-degree relatives, those with frequent noncompliance with ASM therapy, those with only non-motor seizures without impaired consciousness, or those with progressive developmental epileptic encephalopathies. We analyzed the WES of 400 Korean PWEs to identify candidate genes related to epilepsy. The clinical characteristics of the 400 Korean PWEs are presented in Table S2. Our WES data analysis followed a workflow described in our previous study with some modifications (Kang et al., 2022). In brief, of the variants of the known epilepsy-associated genes (*n*=215) that cause pure or relatively pure epilepsies or syndromes with epilepsy as the core symptom (Wang et al., 2017), only those with a read depth over 30× were included. The MAF of the selected variants in the PWEs was compared to that in an East Asian population (gnomAD) or in a Korean population (KRGDB; Jung et al., 2020).

### Fish husbandry

Zebrafish (AB strain) were acquired from the Korea Zebrafish Resource Center, reared under standard protocols in a fish facility and staged at 28.5°C based on standard criteria (Kimmel et al., 1995). Zebrafish under 1 year of age were utilized for the study, and sex was not a selection criterion. For Ca^2+^ imaging experiments, *slc25a22a^+/−^* zebrafish were outcrossed with *Tg(elavl3:Gal4,UAS:GCaMP6s)* zebrafish (Choe et al., 2021) provided by the Fluorescent Reporter Zebrafish Cooperation Center (#1024) in Korea.

### DNA manipulation

Zebrafish *slc25a22a* (GenBank Accession Number XM_001922037.5) was PCR amplified from the complementary DNA (cDNA) synthesized from 3 dpf embryos using a primer pair (Table S2) and then cloned into the NcoI/XbaI sites in pCS4+ vector (Jin et al., 2004). To construct a plasmid encoding GFP fused to the C-terminal of *slc25a22a*, we PCR amplified two DNA fragments from pCS4+-*slc25a22a* and pEGFP-N1 (Clontech) using specific primer pairs (Table S3). The resulting two PCR products were digested with ClaI/EcoRV and EcoRV/XbaI enzymes (New England Biolabs), respectively, and then ligated simultaneously into the ClaI/XbaI sites of the pCS4+ vector. To generate riboprobe, *kcnc1a* (NM_001128725.1), *kcnc1b* (NM_001195197.1), *kcnma1a* (NM_001145600.1), *kcnt1* (XM_009295129.3), *gabrb3* (XM_005166080.4), *chrnb2a* (XM_021476333.1), *chrnb2b* (XM_005169754.4), *pnpo* (NM_001256178.1), *wwox* (NM_200913.1), *slc25a22b* (XM_009302949.3), *slc2a1a* (NM_001039808.2), *slc2a1b* (XM_002662528.5), *gosr2* (NM_199688.1), *depdc5* (XM_686358.8), kctd7 (NM_001045333.2), *szt2* (XM_021476976.1) and *kcnq2a* (NM_001114907.1) were PCR amplified from zebrafish cDNA using gene-specific primers (Table S3) and then individually cloned into pCS4+ vector.

### WISH

Plasmids containing the specified genes were digested with a restriction enzyme (New England Biolabs). These resulting linearized plasmids were then used as templates to create antisense riboprobes, utilizing T7 RNA polymerase (Enzynomics, RP001S) and a DIG RNA Labeling Kit (Sigma-Aldrich, 11277073910). WISH was performed as per standard protocols (Chitramuthu and Bennett, 2013). The resulting images were taken with a stereomicroscope (Leica, MZ-16) and then assembled using Photoshop (Adobe).

### MO injections

MOs targeting either the start codon or the boundaries between exons and introns were obtained from Gene Tools and microinjected into one-cell-stage zebrafish embryos. To test the efficiency of MOs targeting the start codon, two complementary oligonucleotides containing the target sequence of the MOs (see below) were annealed and cloned into the BamHI/NcoI sites of the pCS2+-EGFP vector. We assessed the intensity of GFP fluorescence by co-microinjecting 4 ng MOs and RNAs encoding the MO target fused to GFP at the N-terminus. To assess the efficacy of splicing MOs, reverse transcription PCR (RT-PCR) analysis was performed using RNA isolated from 1 dpf embryos. Prime Taq polymerase (GENETBIO) and specific primers for each target (Table S3) were used. Amplicons were subsequently sequenced.

The following MOs were used: *kcnc1b* MO, 5′-GTACCTCTCTAGTCATATCTCCAAG-3′ (translation blocking MO); *kcnma1a* MO, 5′-TAAGTGAAATGTGTACTTACGCGCA-3′ (splicing blocking MO); *chrnb2a* MO, 5′-TGACAAACACGATGACATACTTGTT-3′ (splicing blocking MO); *chrnb2b* MO, 5′-AGAAGAGCGTCCACAGAGCCATCAT-3′ (translation blocking MO); *slc2a1a* MO, 5′-ACCTCCTTTTTATTAGACTCCATGA-3′ (translation blocking MO); *slc2a1b* MO, 5′-CCTTCCATCACGTCTTGTCAAAATT-3′ (translation blocking MO); *slc25a22a* MO, 5′-GATCTGTTTGTCAGCCATTTATCTG-3′ (translation blocking MO); *slc25a22b* MO, 5′-CTGATCTGATTGTCAGCCATTTCAA-3′ (translation blocking MO); *szt2* MO, 5′-CCGACCTGAACCAAACACATTCATA-3′ (splicing blocking MO); *kctd7* MO, 5′-GCCTCTAATTATTCATATTACCTTT-3′ (splicing blocking MO); *pnpo* MO, 5′-TCTCATGTTACTAAGATCCATGCTG-3′ (translation blocking MO); and *wwox* MO, 5′-TATTTGAGAGCCGCCATTGCGAAAT-3′ (translation blocking MO).

### PTZ treatment

PTZ (P6500) was obtained from Sigma-Aldrich. For the stock solution, PTZ was dissolved in ultrapure water. For treatments, PTZ was diluted in egg water to final concentrations of 1-10 mM and then administered to 5 dpf larvae for 30 min.

### Behavior analysis

Larvae at 5 dpf were placed into either a 48-well plate containing 800 μl egg water [sea salt (0.5 g/l, Instant Ocean) in water] per well or a 96-well plate containing 500 μl egg water. They were transferred to a DanioVision chamber (Noldus), acclimatized for at least 10 min, and recorded for 10 min under normal lighting conditions for 10 min. An automated camera was used to track individual larvae, and the distance traveled and total velocity were quantified using EthoVision XT software 9 or 11.5 (Noldus). When recording locomotor activity, 0.2 mm minimum distance moved was adopted to filter out background noise such as the instrument's own noise.

### Transfection

COS-7 cells were cultured in RPMI-1640 medium (Sigma-Aldrich, R8005) supplemented with 10% fetal bovine serum (Sigma-Aldrich, F2442), 1× GlutaMAX (Gibco, 35050061) and 1× Antibiotic-Antimycotic (Thermo Fisher Scientific, 15240062) at 37°C and 5% CO_2_ atmosphere. pCS4+-*slc25a22a*-EGFP and pMito-DsRed were co-transfected into ∼65-70% confluent COS-7 cells seeded onto a 35 mm confocal glass-bottom dish (SPL Lifesciences, 101350) at a density of 5×10^4^ cells using Lipofectamine 3000 (Thermo Fisher Scientific, L3000001) according to the manufacturer's protocol. At 23-24 h post-transfection, the cells were rinsed thoroughly three times with 500 μl Dulbecco's phosphate buffered saline (DPBS; Welgene, LB001-01) and fixed with 4% paraformaldehyde (Sigma-Aldrich, P6148) at room temperature for 10 min. Subsequently, they were washed three times with DPBS for 2 min each, mounted with Vectashield Plus Antifade Mounting Medium with DAPI (Vector Laboratories, H-2000) and imaged immediately using a confocal laser scanning microscope (Leica, LSM700). Images were acquired by Zen Black (version 8.1; Zeiss), processed with ImageJ BioFormats plugin and assembled using Adobe Illustrator.

### Generation of *Tg(mito:dsred)* zebrafish

The DsRed coding sequence was amplified from pDsRed2 (Clontech) and then cloned into the ClaI/NcoI sites of mini-Tol2-mitochondrial localization sequence (MLS)-EGFP (Kim et al., 2008) to replace EGFP with DsRed. Subsequently, the resulting plasmid and transposase mRNA were co-microinjected into one-cell-stage zebrafish embryos to generate *Tg(mito:dsred)*, as described previously (Fisher et al., 2006).

### CRISPR-Cas9-mediated mutagenesis and genotyping

Two different single-guide RNAs (sgRNAs) targeting the second and third exons of *slc25a22a* (5′-GGCGGUGUUGCCGGACUGAU-3′ and 5′-GGAUACUUUGGCAUGUAUAG-3′, respectively), were designed with ZiFiT (Sander et al., 2007). To generate the *slc25a22a* sgRNAs, we annealed corresponding oligonucleotides (Table S3) and then cloned them into the BsmBI site of the pT7-gRNA vector (Hwang et al., 2013). The resulting construct was digested with BamHI and *in vitro* transcribed with an mMESSAGE mMACHINE T7 Transcription Kit (Invitrogen, AM1344). The plasmid encoding Cas9 optimized for zebrafish codon usage (Jao et al., 2013) was digested with XbaI, and Cas9 RNA was synthesized using an mMESSAGE mMACHINE T3 Transcription Kit (Invitrogen, AM1348). The two different *slc25a22a* sgRNAs (12.5 ng each) and Cas9 RNA (300 ng) were co-microinjected into one-cell-stage zebrafish embryos. Knockout efficiency was validated with PCR using the primers listed in Table S3. For zebrafish genotyping, genomic DNA (gDNA) was extracted from skin cells using the skin swabbing method (Jang et al., 2022) and subjected to PCR with the primers listed in Table S3. The resulting PCR products were analyzed with 1.5% or 4% agarose gel electrophoresis and sequenced at Cosmo Genetech.

### RNA isolation and cDNA synthesis

Total RNA was extracted from 25 larvae at 5 dpf using TRIzol (Molecular Research Center, TR 118). The resulting total RNA (1 μg) was used to synthesized cDNA with oligo(dT) primers and a TOPscript cDNA Synthesis Kit (Enzynomics, EZ005S) as per the manufacturer's instructions.

### Western blotting

Embryos at 5 dpf were decapitated, and their heads were lysed in RIPA buffer (Thermo Fisher Scientific, 89900) with 1× Halt Protease Inhibitor Cocktail (Thermo Fisher Scientific, 78429). After homogenization, 50 µg total proteins were separated with 12% sodium dodecyl sulfate polyacrylamide gel electrophoresis and transferred to a PVDF membrane (Millipore, IPVH10100). To enhance the detection of specific proteins in western blot analysis, we used a Pierce Western Blot Signal Enhancer (Thermo Fisher Scientific, 21050). The primary antibodies used were anti-SLC25A22 antibody (1:500; Thermo Fisher Scientific, PA5-53207) and anti-β-Actin antibody (1:1000; Abcam, ab6276). The secondary antibodies used were horseradish peroxidase (HRP)-conjugated goat anti-rabbit antibody (1:2500; ABclonal, AS014) and HRP-conjugated goat anti-mouse antibody (1:10,000; ABclonal, AS003). The images were captured using ChemiDoc MP (Bio-Rad).

### LFP recording

The electrographic brain activity of 5 dpf larvae was assessed by LFP recordings from the optic tectum as described previously (Baraban, 2013; Zhang et al., 2022) with some modifications. In brief, the zebrafish larvae were pretreated with 0.3 mM pancuronium bromide (Sigma-Aldrich, 15500-66-0) for 10 min. A blunt glass capillary (Harvard Apparatus, 30-0057) was pulled with a micropipette puller (Sutter Instrument, P-1000) with 2 M NaCl (3-5 MΩ) inside and positioned on the skin above the optic tectum of a larva embedded in 2% low-melting point agarose (Thermo Fisher Scientific, 16520050) placed in a recording chamber, which was filled with recording medium (1 mM NaCl, 2.9 mM KCl, 10 mM HEPES, 1.2 mM MgCl_2_, 10 mM dextrose, 2.1 mM CaCl_2_, adjusted to pH 7.3 with 1 N NaOH). Data were collected using an NI USB-6251 data acquisition module (National Instruments), amplified with an amplifier (Axon Instruments, Axopatch 200B), and analyzed with WinEDR (John Dempster, University of Strathclyde). For the drug efficacy experiment, the zebrafish larvae were treated with 300 μM VPA for 2 h and 0.3 mM pancuronium bromide for 10 min and then embedded. Each recording was conducted for 20 min and was visualized with WinEDR. Spikes over 0.2 mV were counted as a seizure-like event.

### Ca^2+^ imaging and data analysis

*Tg(elavl3:Gal4,UAS:GCaMP6s)* larvae at 5 dpf were individually immobilized in a drop of 1% low-melting point agarose with their tails freed from the agarose in a microscope slide (Marienfeld Superior, HSU-1000612) with silicone isolators (Invitrogen, P24740). GCaMP6s signals were captured from the larvae at 5 dpf for 20 min with a fluorescence microscope (Zeiss, SteREO Discovery), and the frequency of GCaMP6s fluorescence traveling from the forebrain to the spinal cord was counted.

### Chemical treatment

VPA (P4543), CBZ (C4024) and TGB (SML0035) were obtained from Sigma-Aldrich, and VGB (HY-15399) was purchased from MedChemExpress. Other ASMs used in this study were provided by Myung-In PHARM. For the stock solution, VPA was dissolved in ultrapure water, and the other ASMs were dissolved in DMSO. All ASMs tested were diluted in egg water to final concentrations of 25-500 µM and then administered to 5 dpf larvae for 2 h.

### Statistics and reproducibility

Data are expressed as mean±s.d., with error bars representing s.d. *P*-values were determined by one-way ANOVA with Tukey's honest significant difference (HSD) post hoc test. Microsoft Excel (version 2016) and GraphPad Prism (version 9.4) were used for statistical analysis. All experiments in this study were repeated independently at least twice with similar results.

## Supplementary Material

10.1242/dmm.052275_sup1 Supplementary information
