# Supplementary material for: Mutant zebrafish lacking slc25a22a show spontaneous seizures and respond to the anti-seizure medication valproic acid
Source: Dis Model Mech. 2025 Jun 20;18(6):dmm052275. doi: 10.1242/dmm.052275 (PMC12208195; doi:10.1242/dmm.052275)
Supplement: Supplementary information [file dmm-18-052275-s1.pdf]

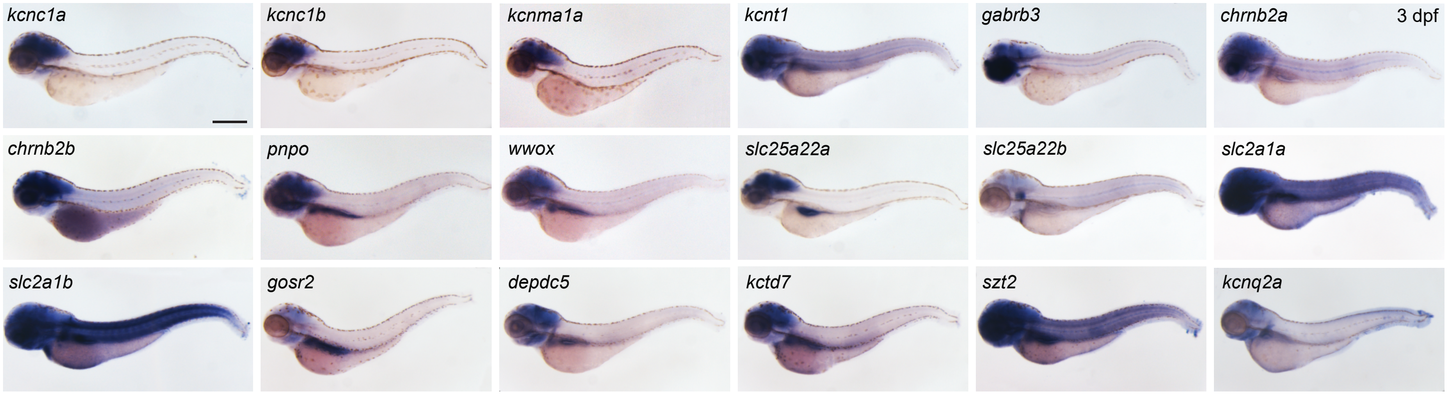

Fig. S1. Candidate genes in Korean patients with epilepsy are expressed in the brain.

WT zebrafish larvae at 3 dpf were probed with *kcnc1a*, *kcnc1b*, *kcnma1a*, *kcnt1*, *gabrb3*, *chrnb2a*, *chrnb2b*, *pnpo*, *wwox*, *slc25a22a*, *slc25a22b*, *slc2a1a*, *slc2a1b*, *gosr2*, *depdc5*, *kctd7*, *szt2* and *kcnq2a* riboprobes. Lateral view anterior to the left. Scale bar = 300 µm.

Disease Models & Mechanisms • Supplementary information

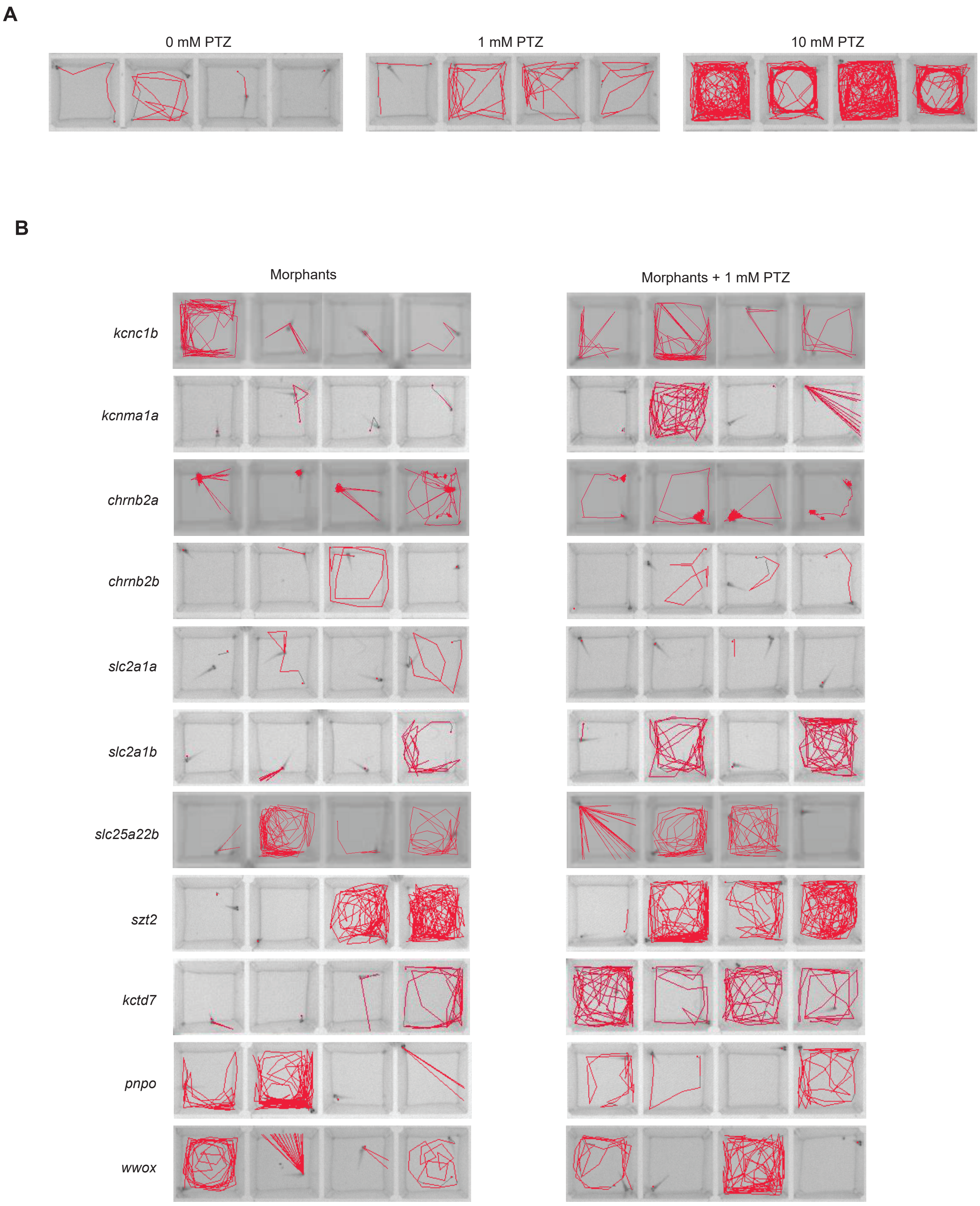

Fig. S2. Epilepsy related gene morphants exhibit no significant abnormal phenotype, excluding *slc25a22a* and *slc25a22b*

(A) Representative locomotor activity traces of WT larvae treated with 0, 1, and 10 mM pentylenetetrazol (PTZ) at 5 dpf for 10 min. **(B)** Representative locomotor activity traces recorded for 10 min of *kcnc1b*, *kcnma1a*, *chrnb2a*, *chrnb2b*, *slc2a1a*, *slc2a1b*, *slc25a22b, szt2, kctd7, pnpo* and *wwox* morphants in the absence or presence of 1 mM PTZ at 5 dpf.

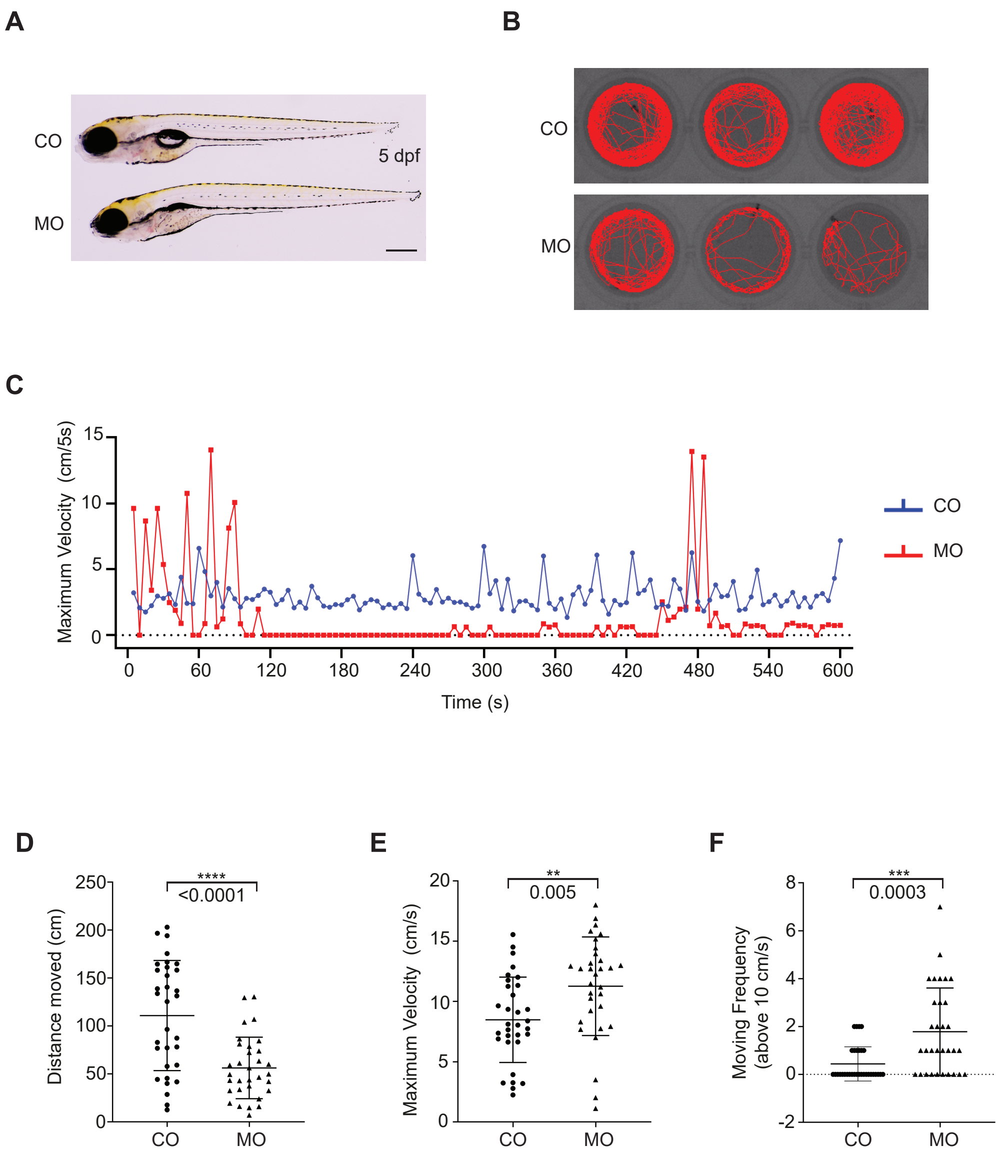

Fig. S3. ***slc25a22a*** **morphants display spontaneous seizures.**

One-cell stage zebrafish embryos were microinjected with antisense oligonucleotides morpholinos (MOs) targeting *slc25a22a*, exposed to 1 mM PTZ at 5 dpf for 30 min, imaged under a light microscope (lateral view anterior to the left; scale bar = 200 µm) **(A)**, assessed for locomotor activity and swim maximum velocity (cm/5s) for 10 min **(B, C)**, and evaluated for total swimming distance, swimming maximum velocity and moving frequency (over 10 cm/s) for 10 min **(C, D, E)**. Data are presented as mean ± SD. $^{**}P < 0.01$, $^{***}P < 0.001$ and $^{****}P < 0.0001$ by one-way ANOVA with Tukey's honest significant difference (HSD) post hoc test ($n = 32$ embryos per each group). CO: control.

Disease Models & Mechanisms • Supplementary information

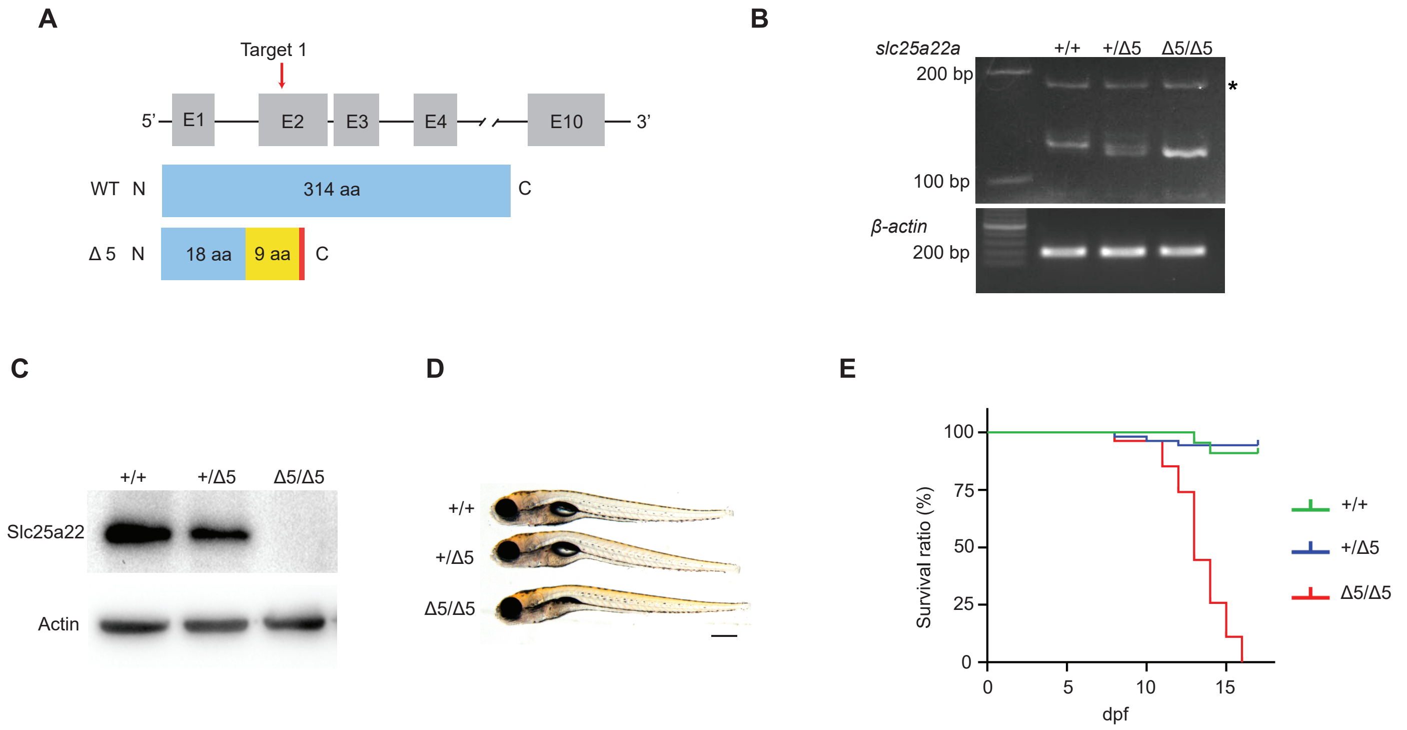

Fig. S4. *slc25a22a*$^{\Delta5/\Delta5}$ mutants is generated with CRISPR-Cas9 technology.

(A) Upper: schematic of the zebrafish *slc25a22a* locus and location of CRISPR/Cas9 targets (red arrow); lower: schematic of Slc25a22a proteins translated in WT and *slc25a22a*$^{\Delta5/\Delta5}$ mutants generated with the CRISPR-Cas9 technology. The blue and yellow boxes indicate WT and introduced amino acids, respectively, and the red bar represents a premature stop codon. **(B)** RT-PCR analysis of the targeted *slc25a22a* region in indicated alleles. mRNAs were harvested from zebrafish larvae at 5 dpf and amplified with gene-specific primers. β-actin primers were used for loading control. Asterisk represents non-specific amplification. **(C)** Western blotting analysis of Slc25a22 proteins in 5-dpf larvae with indicated alleles. Actin protein was used as loading control. **(D)** Larvae with indicated alleles at 5 dpf were imaged under a light microscope. Lateral view anterior to the left. Scale bar = 200 µm. **(E)** *slc25a22a*$^{+/+}$ (n = 22), *slc25a22a*$^{+/\Delta5}$ (n = 55) and *slc25a22a*$^{\Delta5/\Delta5}$ (n = 16) embryos were raised to 17 dpf and their respective Kaplan-Meier survival rate curves were generated.

Disease Models & Mechanisms • Supplementary information

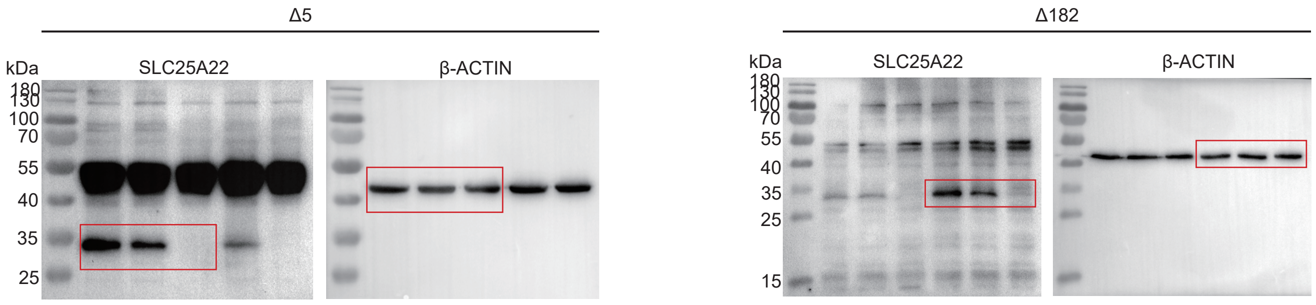

Fig. S5. Uncropped Western blots for the main figure 3B.

Disease Models & Mechanisms • Supplementary information

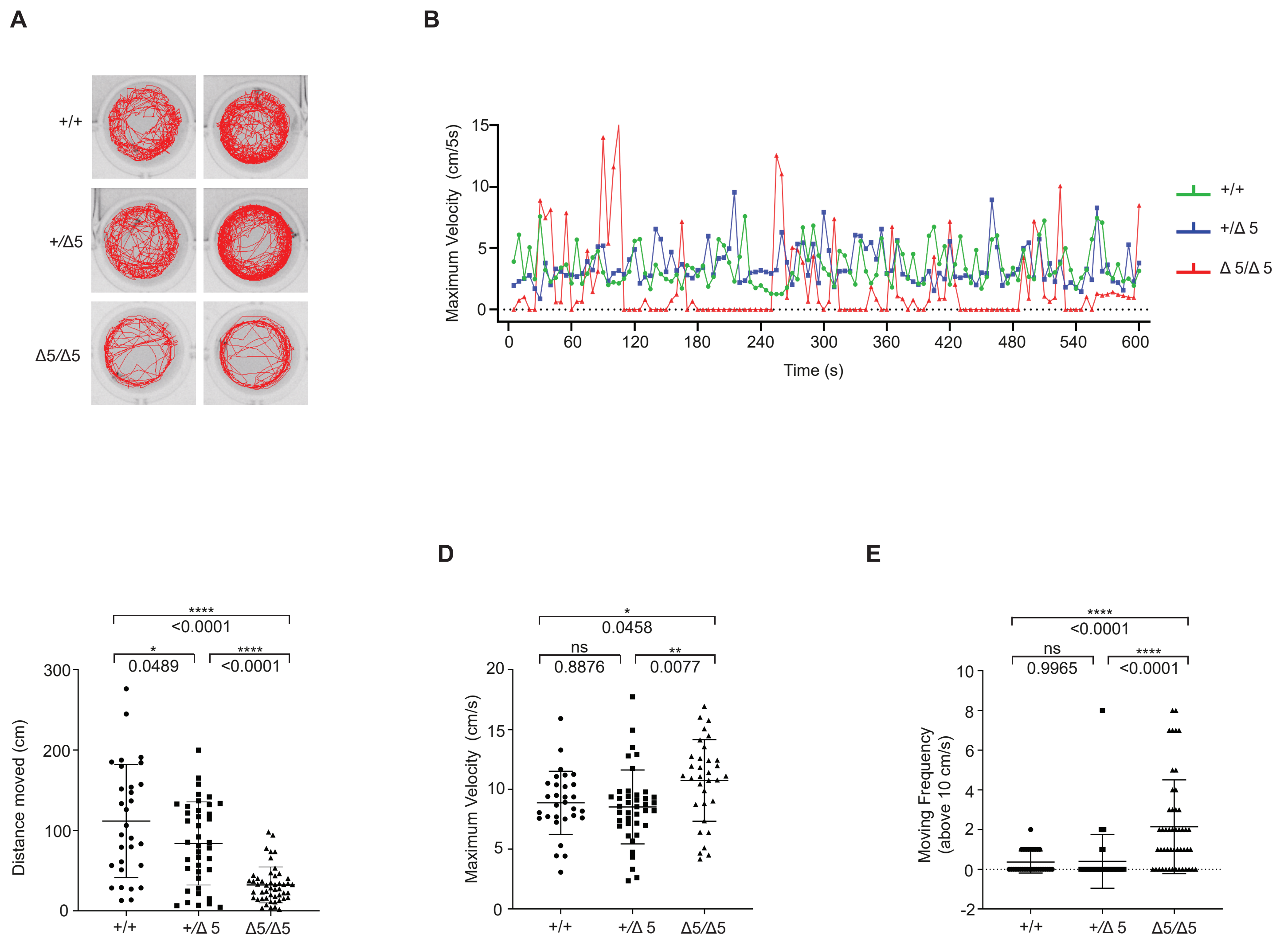

Fig. S6. *Slc25a22a*$^{\Delta5/\Delta5}$ mutants display spontaneous seizures.

(A) Representative locomotor activity traces of *slc25a22a*$^{+/+}$, *slc25a22a*$^{+/\Delta5}$ and *slc25a22a*$^{\Delta5/\Delta5}$ larvae at 5 dpf for 10 min. **(B)** Representative traces of swim maximum velocity (cm/5s) in *slc25a22a*$^{+/+}$ (green), *slc25a22a*$^{+/\Delta5}$ (blue), and *slc25a22a*$^{\Delta5/\Delta5}$ (red) larvae at 5 dpf for 10 min. **(C, D, E)** The total swimming distance, swimming maximum velocity, and moving frequency (over 10 cm/s) assessed for 10 min in *slc25a22a*$^{+/+}$, *slc25a22a*$^{+/\Delta5}$, and *slc25a22a*$^{\Delta5/\Delta5}$ larvae at 5 dpf. Data are presented as mean ± SD. *$P < 0.05$, **$P < 0.01$ and ****$P < 0.0001$ by one-way ANOVA with Tukey's HSD post hoc test ($n = 40$ embryos per each group).

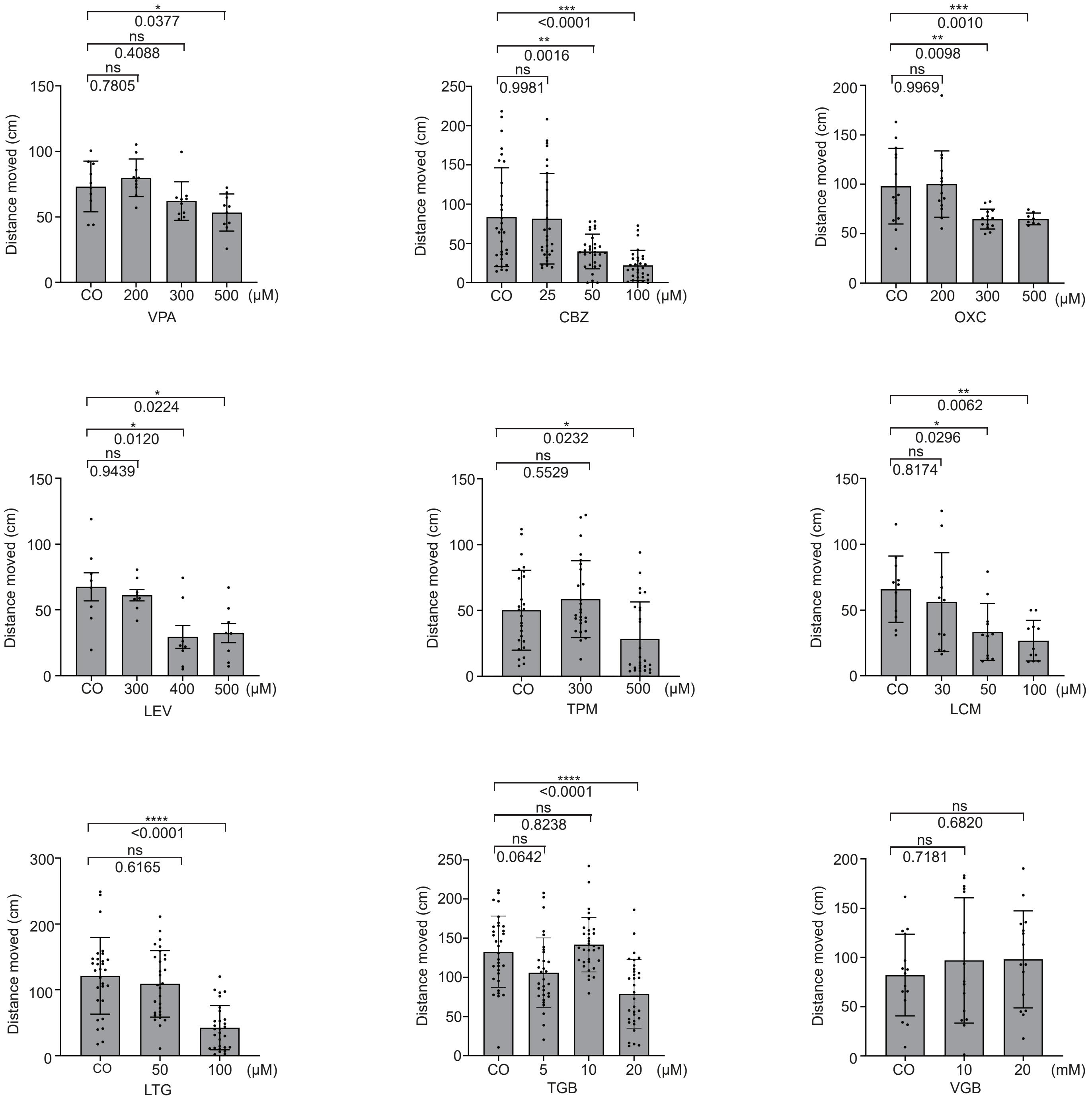

Fig. S7. Determination of the highest non-toxic concentrations of ASDs in WT zebrafish larvae.

WT zebrafish larvae at 5 dpf were exposed to the indicated ASDs and their total swimming distances were assessed. Data are presented as mean ± SD. *$P < 0.05$, **$P < 0.01$, ***$P < 0.001$ and ****$P < 0.0001$ by one-way ANOVA with Tukey's HSD post hoc test (VPA: $n = 10$; CBZ: $n = 30$; OXC: $n = 14$; LEV: $n = 8$; TPM: $n = 26$, LCM: $n = 11$; LTG: $n = 29$: TGB: $n = 32$; VGB: $n = 15$).

Table S1. Eighteen candidate genes shortlisted from the whole exome sequencing analysis.

Human	Zebrafish
KCNC1	*kcnc1a*
	kcnc1b
KCNMA1	*kcnma1a*
KCNT1	*kcnt1*
CHRNB2	*chrnb2a*
	chrnb2b
PNPO	*pnpo*
WWOX	*wwox*
SLC25A22	*slc25a22a*
	slc25a22b
SLC2A1	*slc2a1a*
	slc2a1b
GOSR2	*gosr2*
KCTD7	*kctd7*
SZT2	*szt2*
KCNG2	*kcnq2*
GABRB3	*gabrb3*
DEPDC5	*depdc5*

Table S2. Characteristics of the 400 Korean patients with epilepsy.

Age (years)		
At recruitment		39.6 ± 13.5 (range: 19~84)
At seizure onset		20.7 ± 14.1 (range: 0~68)
Gender (male) *n* (%)		210 (52.5)
Epilepsy classification, *n* (%)		
Generalized		27 (6.8)
Focal		373 (93.2)
Brain imaging, *n* (%)		
Not available		34 (8.5)
Normal finding		197 (49.3)
Epilepsy-relevant lesion		169 (42.3)
	Hippocampal sclerosis	43
	Posttraumatic lesions	36
	Focal cortical dysplasia	17
	Stroke	16
	Infection	14
	vascular malformation	10
	Tumor	7
	Others	26

Disease Models & Mechanisms • Supplementary information

Table S3. List of primers used in the study.

Name	Sequences
slc25a22a CDS	Forward: TAG TCC CAT GGA CCA TGG CTG ACA AAC AGA TCA
	Reverse: CGC GAT CTA GAT TAG TTG TTT CGT TTG GGT AGA
slc25a22a sgRNA 1	Forward: TAG GCG GTG TTG CCG GAC TGA T
	Reverse: AAA CAT CAG TCC GGC AAC ACC G
slc25a22a sgRNA 2	Forward: TAG GAT ACT TTG GCA TGT ATA G
	Reverse: AAA CCT ATA CAT GCC AAA GTA T
slc25a22a Δ5 genotyping	Forward: CTT CAG TTT GCC TGC CAA GC
	Reverse: CAT TCT GCT GGT TCT GAA GG C
slc25a22a Δ182 genotyping	Forward: CTC GTA GCC ATC TTA CAG ACA G
	Reverse: CTA TGT TGG ATC TTA CGG GC
slc25a22a-EGFP	Forward: GCA TAT ATC GAT ACC ATG GCT GAC AAA CAG ATC AG
	Reverse: GCG CAT GAT ATC GTT GTT TCG TTT GGG TAG ATG
	Forward: ATA TAT GAT ATC CCG CGG GCC CGG GAT CCA CC
	Reverse: ATA TAT TCT AGA TTA CTT GTA CAG CTC GTC C
Tg(mito:dsred)	Forward: GAC CGG TCG CCA CCA TGG CCT CCT CCG AGA ACG
	Reverse: TGG ATC ATC ATC GAT CTA CAG GAA CAG GTG GTG G
kcnc1a	Forward: TAG TCG AAT TCA CCA TGG GCC AGG GCG ACG AG
	Reverse: GGA TAC TCG AGT CAG GTG GTG ACC CTG GCA T
kcnc1b	Forward: TAG TCG GAT CCA CCA TGA CCT ACC GGC AG
	Reverse: TAG TAC TCG AGC TAC AGG ATG GAT GGG AT
kcnma1a	Forward: TAG TCG GAT CCC TAT AGT GCG GTA AAT GG
	Reverse: TAC TAC TCG AGG GGG AAG TTG TGA AGG GTT
kcnt1	Forward: TAG TCG GAT CCA TCT GTG GAT GGA AAG AC
	Reverse: TAG TCC TCG AGT GTA ATA GGC TCT TAA AGG C
gabrb3	Forward: TAG TCG AAT TCA CCA TGC GCC TTG CCG GGT GG
	Reverse: CGC GAC TCG AGC TAA TTC ACA TAA TAC AGC C
chrnb2a	Forward: TAG TCG AAT TCA CCA TGA CAA CAA ATG TCT GGC
	Reverse: TAG TCC TCG AGT CAC TCT TCC TCT GCG A
chrnb2b	Forward: TCG CGG GAT CCA CCA TGA AAA TGC TAA TTA AG
	Reverse: TAG TCC TCG AGT CAG CCT GGT GTG TGG GTG A
pnpo	Forward: TAG TCG GAT CCA CCA TGG ATC TTA GTA ACA TG
	Reverse: TAG TCG AAT TCT CAA GGG GAC AAG CGC T
wwox	Forward: TAG TCG GAT CCA CCA TGG CGG CTC TCA AAT AC
	Reverse: TAG TCG AAT TCC ATG GAC TTG GTG AAG GGT CTC
slc25a22b	Forward: TAG TCG GAT CCA CCA TGG CTG ACA ATC AGA

Disease Models & Mechanisms • Supplementary information

	Reverse: TAG TCC TCG AGT CAG TGT ATT TGT CCG TG
slc2a1a	Forward: TCG CGG GAT CCA CCA TGG AGT CTA ATA AAA AGG<br>Reverse: TAG TCC TCG AGT CAG AGT TGT GAA TCT
slc2a1b	Forward: TAG TCA TCG ATA CCA TGG AAG GCG GAA AGC AAT T<br>Reverse: TAG TCC TCG AGT TAT AGC TGA GAG TCC GC
gosr2	Forward: TAG TCG GAT CCA CCA TGG AGA CGC TTT ACC AT<br>Reverse: TAG TCC TCG AGT CAG CTA AGG TAT TTC AC
depdc5	Forward: TAG TCG GAT CCA CCA TGG TAG AAT CTC AAA GG<br>Reverse: TAG TCG AAT TCT CAA GGG GCA CTC GCG TTC AT
kctd7	Forward: TAG TCG GAT CCA CCA TGC AGC ATA ATG GAG CA<br>Reverse: CGC GAC CAT GGT CAC CAC CAG GTG ATT TTA A
szt2	Forward: GAC TAG GAT CCC AGT ATT TCA AGA CCG TG<br>Reverse: GAC TAC TCG AGT CGC TGG CAG AGT CTG GA
kcnq2a	Forward: TAG TCG GAT CCA CCA TGG TGC AGA AAT CCC GCA A<br>Reverse: TAG TCC TCG AGT CAA AAC AGT GAG AGA CGC T
kcnma1a MO RT-PCR	Forward: TCA GGC TGA TGG CAC AAC AAA G<br>Reverse: CCA ATA TGA GAG CGG TTG AG
chrnb2a MO RT-PCR	Forward: TTG GCA TCA AAG TCT CTC TCG C<br>Reverse: ATC ACC ATG GCC ACA TAC TTC C
szt2 MO RT-PCR	Forward: GCT GGA CTG TAT GGA TTT GGA G<br>Reverse: ACC TCA ACC CAT GTT GTG CGT T
kctd7 MO RT-PCR	Forward: AGA AGA GGA GTA CCG CAA ACC A<br>Reverse: CTC TGC CAG ATC GCT TAC TAT GCA

Disease Models & Mechanisms • Supplementary information

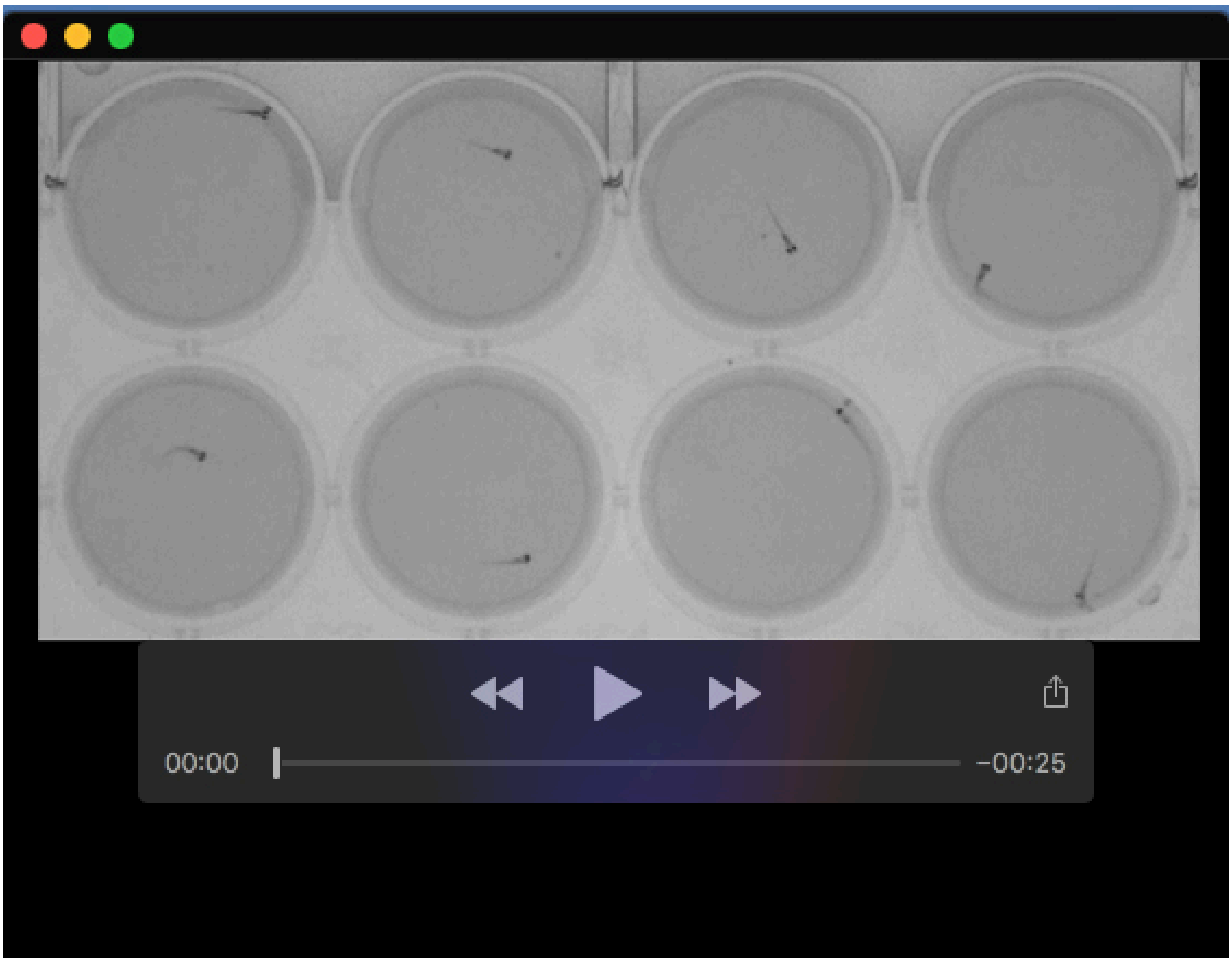

Movie 1. Swimming behavior of WT (upper) and *slc25a22a*$^{\Delta 182/\Delta 182}$ larvae (lower) at 5 dpf.

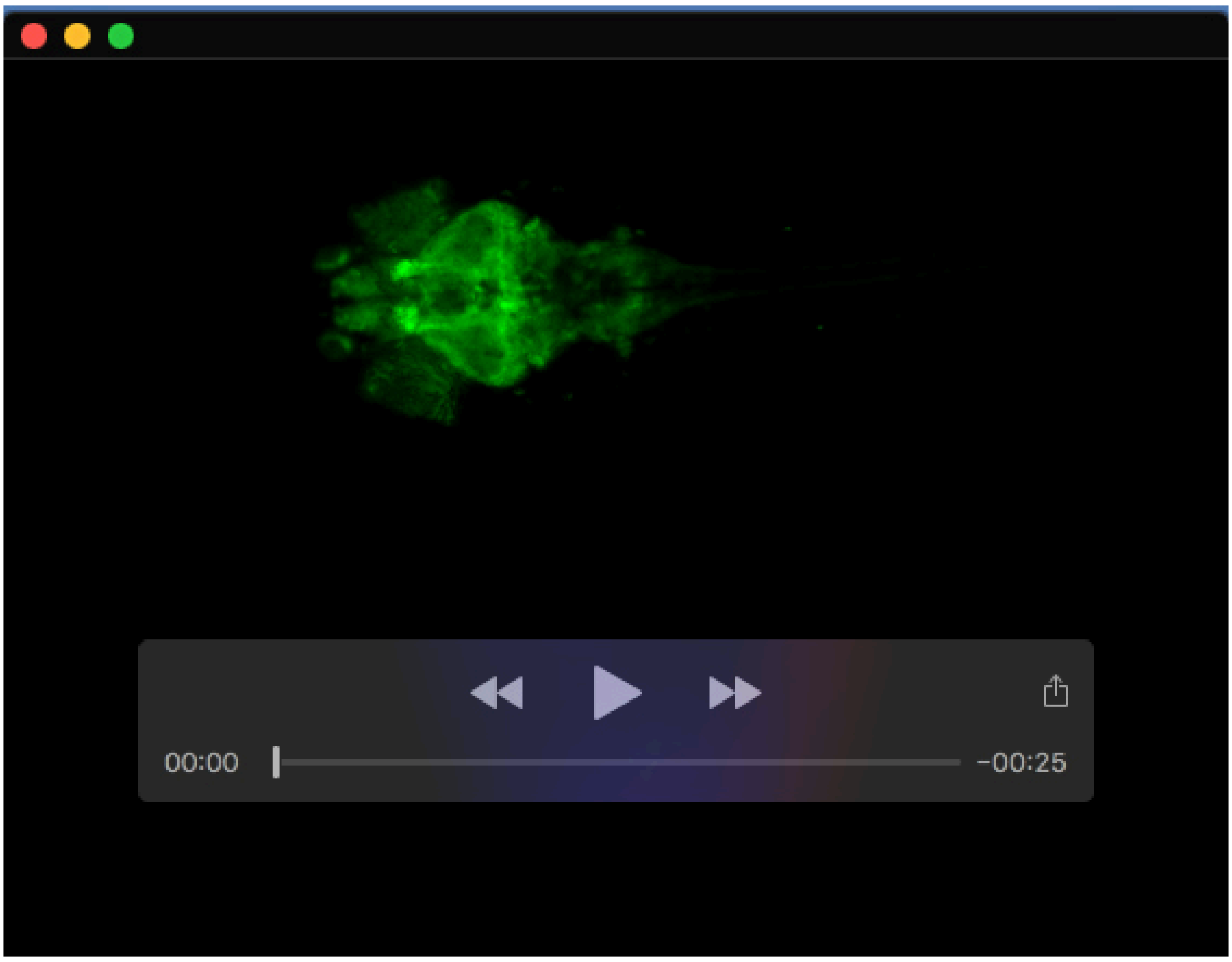

Movie 2. Real-time fluorescence imaging of *slc25a22*$^{+/+}$;*Tg(elavl3:Gal4,UAS:GCaMP6s)* larvae at 5 dpf.

Disease Models & Mechanisms • Supplementary information

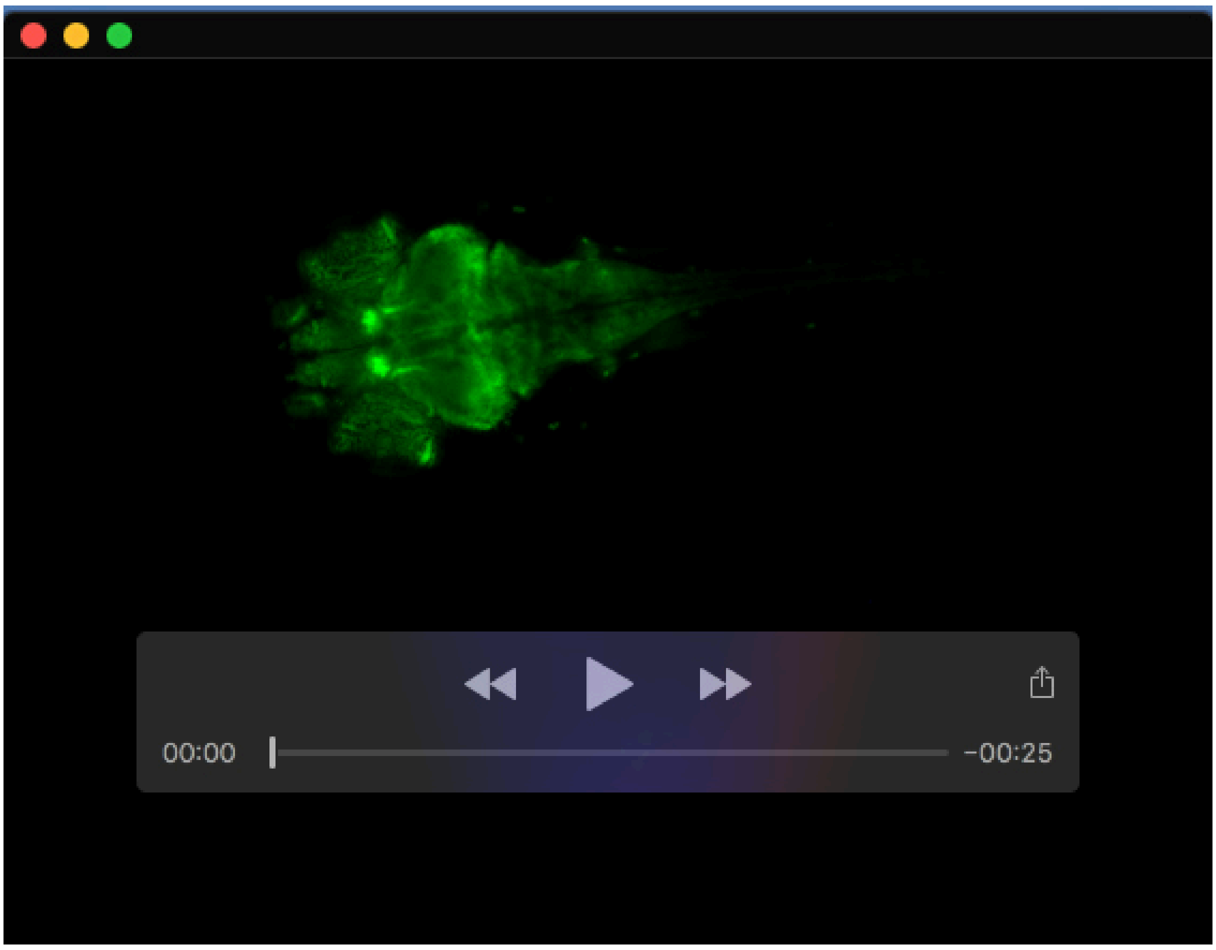

Movie 3. Real-time fluorescence imaging of *slc25a22*$^{+/\Delta182}$;***Tg(elavl3:Gal4,UAS:GCaMP6s)*** larvae at 5 dpf.

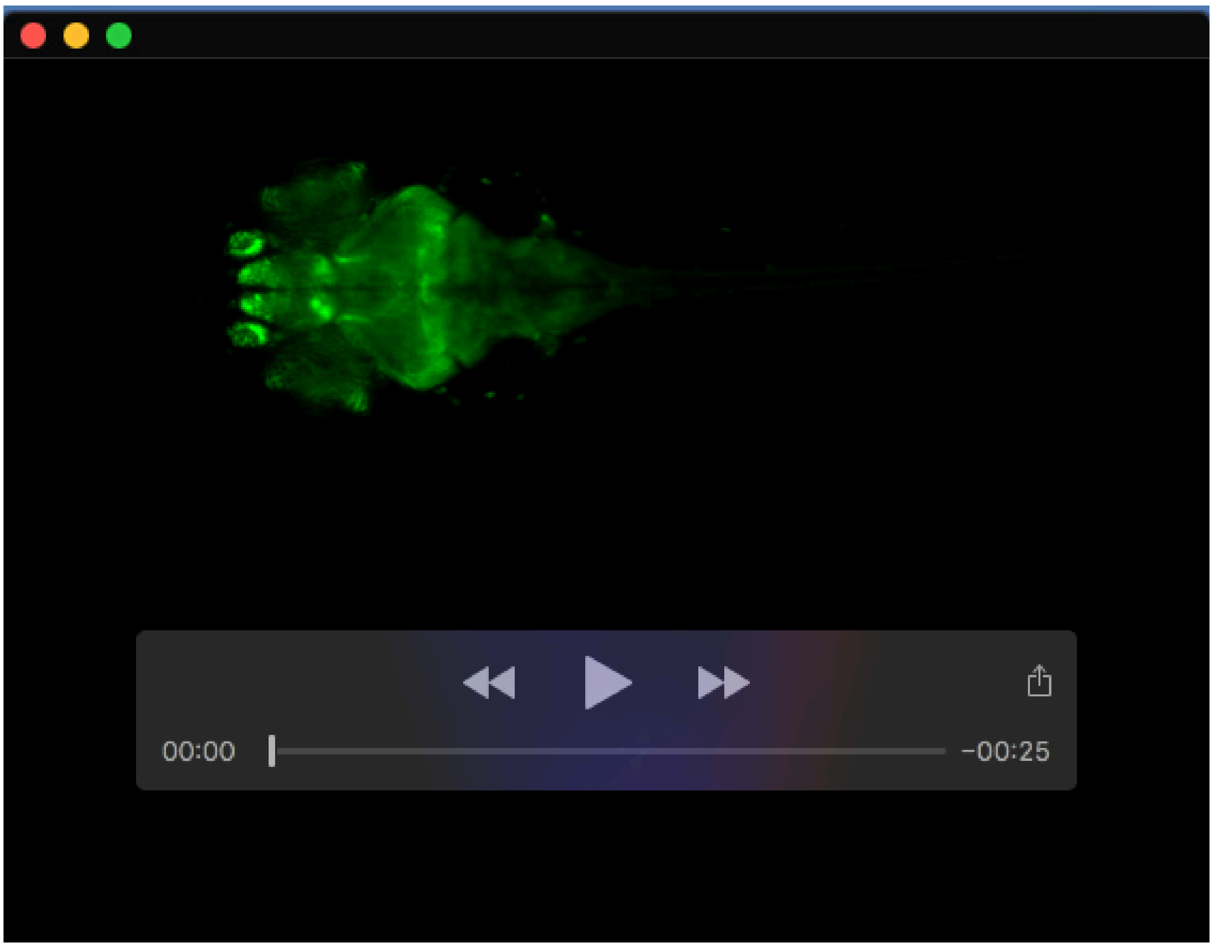

Movie 4. Real-time fluorescence imaging of *slc25a22*$^{\Delta182/\Delta182}$; ***Tg(elavl3:Gal4,UAS:GCaMP6s)*** larvae at 5 dpf.

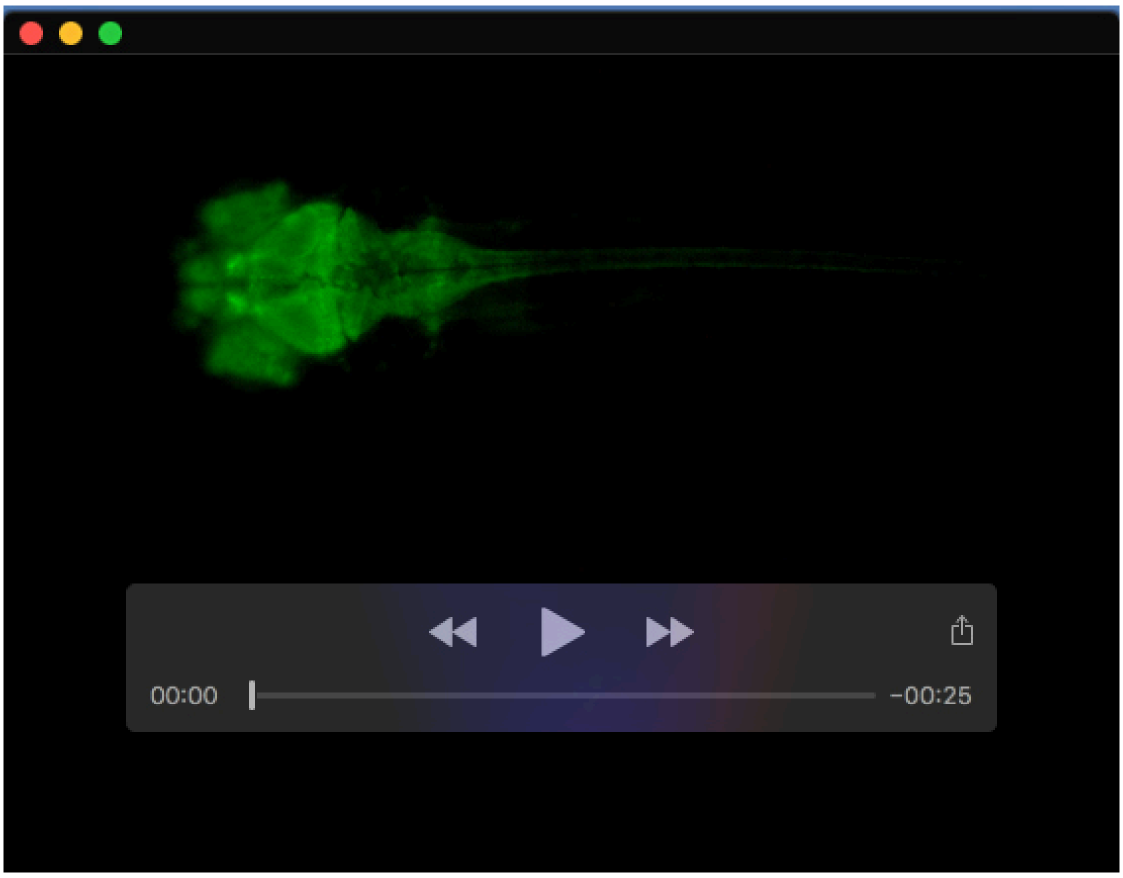

Movie 5. Real-time fluorescence imaging of *slc25a22*$^{+/+}$; ***Tg(elavl3:Gal4,UAS:GCaMP6s)*** larvae treated with PTZ at 5 dpf.

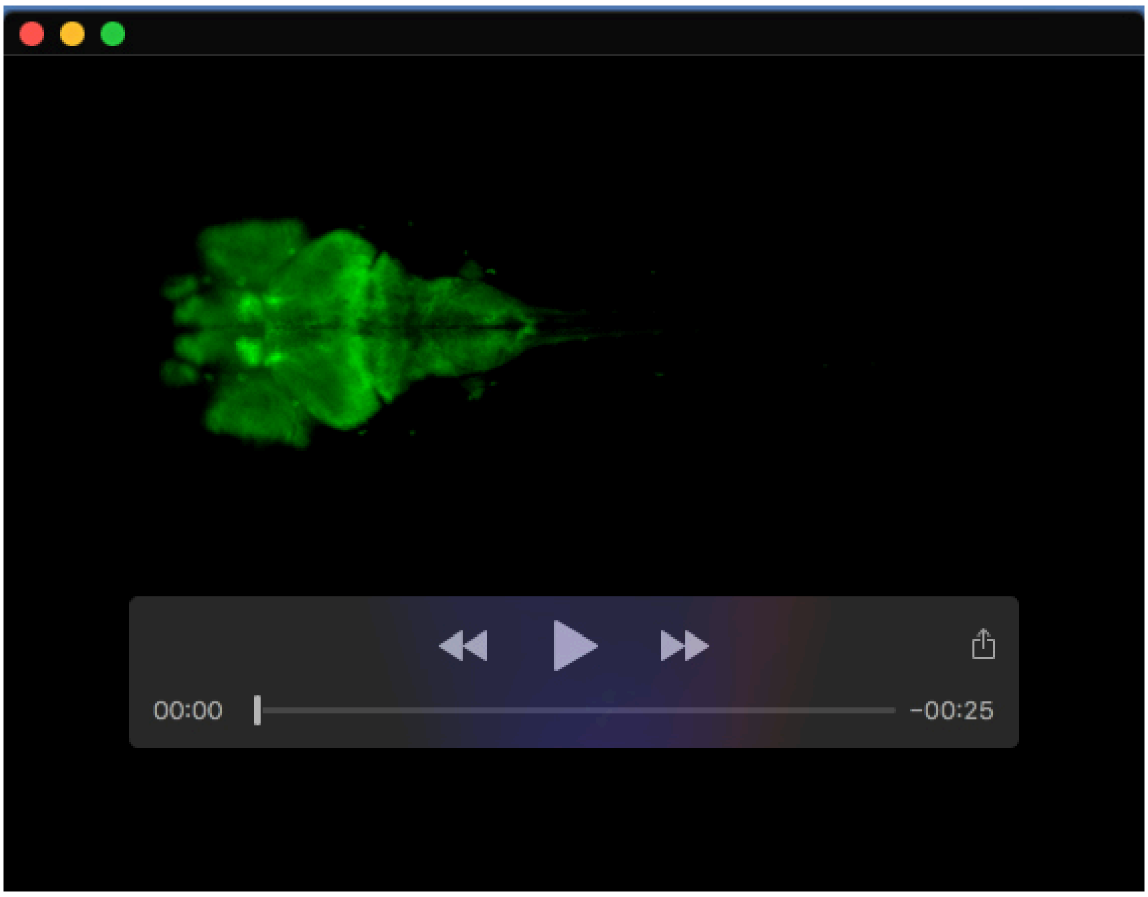

Movie 6. Real-time fluorescence imaging of *slc25a22a*$^{\Delta 182/\Delta 182}$; ***Tg(elavl3:Gal4,UAS:GCaMP6s)*** larvae at 5 dpf treated with VPA.

Disease Models & Mechanisms • Supplementary information
